# Spatiotemporal dynamics and phylogeography of HCoV-NL63 and HCoV-OC43 in Thailand, 2024–2025

**DOI:** 10.1371/journal.pone.0357483

**Published:** 2026-09-08

**Authors:** Jiratchaya Puenpa, Ratchadawan Aeemjinda, Preeyaporn Vichaiwattana, Sumeth Korkong, Yong Poovorawan

**Affiliations:** 1 Department of Pediatrics, Faculty of Medicine, Center of Excellence in Clinical Virology, Chulalongkorn University, Bangkok, Thailand; 2 Division of Research Affairs, Faculty of Medicine, Chulalongkorn University, Bangkok, Thailand; 3 FRS(T), The Royal Society of Thailand, Bangkok, Thailand; Cairo University Faculty of Veterinary Medicine, EGYPT

## Abstract

Endemic human coronaviruses (HCoVs) HCoV-NL63 and HCoV-OC43 are common causes of acute respiratory infections (ARI), yet integrated surveillance and genomic data from Southeast Asia remain limited. We characterized HCoV-NL63 and HCoV-OC43 circulation in Thailand, during 2024–2025 using routine real-time RT-PCR testing, partial spike sequencing, and time-scaled phylogenetic analyses with global references. Among 11,709 ARI specimens, 329/8,122 were HCoV-positive in 2024 (4.05%) and 131/3,587 in 2025 (3.65%). Positivity was strongly seasonal, peaking in winter, and SARS-CoV-2 surges in the same testing stream generally coincided with lower endemic HCoV positivity. Genotype composition differed by virus: HCoV-OC43 was dominated by genotypes K and J at near-equal frequencies (48.3% and 47.2%), whereas HCoV-NL63 was mainly genotype C4 (43.6%), followed by B2 (32.7%) and C3 (20.9%). Time-scaled phylogenies placed Thai sequences across multiple regions of global diversity, consistent with repeated introductions and onward transmission within several co-circulating lineages. Estimated substitution rates were 3.86 × 10^-4^ substitutions/site/year for HCoV-NL63 and 9.27 × 10^-4^ for HCoV-OC43. Discrete-trait phylogeography supported bidirectional connectivity involving Thailand, with virus-specific differences in the most supported routes. Skygrid reconstructions suggested declines in genetic diversity after 2020, overlapping the COVID-19 era, with a more pronounced decrease for HCoV-OC43. Evidence for selection was limited and inconsistent for HCoV-NL63, whereas several HCoV-OC43 sites overlapped codon-based signals of diversifying selection. Overall, these findings provide a baseline for endemic HCoV seasonality, genotype composition, and connectivity in Thailand, and support continued genomic surveillance in Southeast Asia.

## Introduction

Human coronaviruses (HCoVs) belong to the family *Coronaviridae* and are classified within the subfamily *Orthocoronavirinae*, which is divided into four genera: *Alphacoronavirus*, *Betacoronavirus*, *Gammacoronavirus*, and *Deltacoronavirus* [1]. The endemic HCoVs include two alphacoronaviruses (HCoV-229E and HCoV-NL63) and two betacoronaviruses (HCoV-OC43 and HCoV-HKU1). In this study, we focus on HCoV-NL63 and HCoV-OC43. Coronaviruses possess a single-stranded, positive-sense RNA genome that is among the largest of RNA viruses, typically about 22–36 kb. The genomic RNA contains a 5′ cap and a 3′ polyadenylate tail, enabling direct translation of the replicase polyproteins encoded by ORF1a and ORF1b, followed by downstream expression of structural genes [2]. Because spike (S) mediates host cell entry and is a major immune target, S-gene sequences are widely used to place local strains within global phylogenetic frameworks and to track genetic diversity when whole-genome sequencing is not available [3–5].

Among the four endemic HCoVs that circulate globally (OC43, NL63, 229E, and HKU1), OC43 and NL63 contribute substantially to acute respiratory infections (ARI) worldwide and exhibit pronounced temporal and genetic turnover [6,7]. Although severe outcomes are most often reported in high-risk groups, endemic HCoVs have also been detected in severe ARI and fatal cases among previously healthy adults [8]. These results suggest that endemic HCoVs can be associated with clinically relevant illness and argue for continued molecular surveillance. In temperate regions, detections typically rise in winter. In tropical settings, the timing is more variable and still not well resolved across locations and years [9,10]. A practical limitation in Southeast Asia, including Thailand, is the scarcity of publicly available endemic HCoV sequences. This gap makes it harder to define baseline expectations for seasonality and lineage turnover and to place Thai viruses within the broader global diversity [11].

HCoV-OC43 is typically grouped into genotypes A–K using spike (S) phylogenies, while genome-wide evidence supports contributions from both lineage turnover and recombination to OC43 diversification [7]. Genotype A corresponds to the earliest prototype strains reported in the 1960s. Additional genotypes were described more frequently in the 2000s–2010s, including several lineages with evidence of recombination [12–15]. For HCoV-NL63, spike-based phylogenies commonly group viruses into genotypes A–C, with genotype C often resolved into subclades C1–C3 [16,17].

Combining routine detection data with sequence information makes it possible to follow endemic HCoVs through time and to place Thai viruses alongside globally sampled lineages. A discrete-trait phylogeographic analysis then helps distinguish repeated importations from more persistent local transmission, by showing where Thai sequences sit on the global tree and which transitions into or out of Thailand are most strongly supported. However, Thailand has historically contributed relatively few publicly available sequences for endemic HCoVs, limiting robust inference on external sources and Thailand’s connectivity to global lineages [18–20]. Post–COVID-19, expanding genomic surveillance for endemic respiratory viruses can provide baseline expectations for seasonality and evolution and help interpret atypical surges or clusters.

While aggregate endemic HCoV positivity for 2024 has been summarized previously [21], the present study extends the surveillance period to 2024–2025 and adds sequence-based lineage characterization and phylogeographic inference. We investigated the temporal dynamics and phylogeography of HCoV-NL63 and HCoV-OC43 among ARI patients in Thailand, during 2024–2025. We combined routine surveillance with partial spike sequencing and time-resolved phylogenetic analyses against globally sampled references. We aimed to describe seasonal detection patterns, characterize genetic diversity and lineage composition, and infer broad-scale connectivity using discrete regional states. We also used Markov jump–based summaries to describe transitions into and out of Thailand within this framework. Together, these analyses provide baseline genomic evidence for endemic HCoVs in Thailand in the post–COVID-19 period and support comparisons with regional and global circulation patterns.

## Materials and methods

### Ethical statement

This study was reviewed and approved by the Institutional Review Board of the Faculty of Medicine, Chulalongkorn University, Thailand (IRB0977/67) and was conducted in accordance with the principles of the Declaration of Helsinki. We performed a retrospective review of nasopharyngeal swab specimens and associated clinical records that had been collected as part of routine care (January 2024–February 2025). After IRB approval (28 February 2025), residual specimens were additionally included prospectively (March–December 2025). The research data were accessed on 04/03/2025. Because the study involved retrospective, de-identified data and no direct contact with participants, the IRB granted a waiver of informed consent. Age and sex were obtained from the corresponding medical records. All analyses were conducted using anonymized datasets, and no personally identifiable information was available to the study team.

### Surveillance testing for endemic human coronaviruses by real-time RT-PCR

During 2024–2025, we analyzed 11,709 hospitalized acute respiratory infection (ARI) specimens collected at a collaborating tertiary-care hospital in Bangkok, Thailand, as part of an ongoing respiratory virus surveillance program. ARI was defined as a body temperature ≥38°C accompanied by cough, with symptom onset within the preceding 10 days. Patients of all ages presenting with ARI who were hospitalized were eligible for enrollment. Nasopharyngeal swabs were collected in universal transport medium and transported to our center and processed using a routine diagnostic workflow that screens a broad panel of respiratory pathogens by virus-specific real-time RT-PCR assays (e.g., influenza A/B, RSV, SARS-CoV-2, parainfluenza viruses [PIV1–4], adenovirus, and rhinovirus). In this analysis, we focused on endemic human coronaviruses to describe detection frequency, lineage turnover, and phylogeographic patterns. Aggregate endemic HCoV percent positivity for 2024 from this surveillance stream has been reported previously [21]. This study extends the surveillance period to include 2025 and adds partial spike sequencing with time-scaled phylogenetic and phylogeographic analyses.

Nucleic acids were extracted from 200 μL of sample supernatant using the magLEAD 12gC automated system (Precision System Science, Chiba, Japan) according to the manufacturer’s instructions. Endemic human coronaviruses were screened by real-time RT-PCR targeting a conserved ORF1ab region using published primers and probes [22].

SARS-CoV-2 testing was conducted routinely within the same surveillance program throughout 2024–2025 using established real-time RT-PCR methods, and results for 2024 have been reported previously [23]. In this study, SARS-CoV-2 positivity is presented as contextual surveillance information for comparison with endemic HCoV temporal patterns, whereas genomic analyses focus on endemic HCoVs.

### PCR amplification of partial spike regions for HCoV-NL63 and HCoV-OC43

For partial spike gene sequencing, both HCoV-NL63 and HCoV-OC43-positive specimens with a real-time RT-PCR Ct value < 30 (indicating sufficient viral load for reliable sequencing) were selected for sequencing. Of 154 HCoV-NL63–positive and 163 HCoV-OC43–positive specimens identified by real-time RT-PCR, 110 and 89 specimens, respectively, met the Ct < 30 threshold and were selected for sequencing; all were successfully sequenced, with no sequencing failures. Specimens with Ct ≥ 30 were excluded due to insufficient viral load for reliable amplification and were not eligible for sequencing (S1 Fig in S1 File). Partial spike fragments were amplified using a one-step RT-PCR approach. Primer sequences and target amplicons are provided in S1 Table in S1 File.

This approach yielded amplicons of 1,875 nucleotides for HCoV-NL63 and 1,842 nucleotides for HCoV-OC43. Based on alignment to reference spike protein domains, the HCoV-NL63 amplicon (encoding residues <1–624) spans the C-terminal portion of the S1 subunit, the S1/S2 cleavage region, and the N-terminal portion of the S2 subunit, including the fusion peptide and internal fusion peptide. The HCoV-OC43 amplicon (encoding residues 6–611) spans the N-terminal domain and the majority of the receptor-binding domain (RBD) of the S1 subunit, including the receptor-binding motif.

Reactions were set up in 25 μL volumes containing 2–3 μL RNA template (approximately 100 ng to 1 μg), 0.5 μM of each primer, 12.5 μL of 2 × Reaction Mix (0.4 mM of each dNTP and 3.2 mM MgSO_4_), 1 μL SuperScript III RT/Platinum Taq High Fidelity enzyme mix, and nuclease-free water (SuperScript III One-Step RT-PCR System with Platinum Taq High Fidelity; Invitrogen, Carlsbad, CA, USA), following the manufacturer’s instructions. Thermocycling conditions consisted of reverse transcription at 45 °C for 30 min, followed by 40 cycles of 95 °C for 30 s, 52 °C for 30 s, and 68 °C for 1 min 45 s, with a final extension at 68 °C for 5 min. Amplicons were subjected to bidirectional Sanger sequencing using the corresponding forward and reverse primers at First BASE Laboratories Sdn Bhd (Selangor Darul Ehsan, Malaysia).

### Recombination screening

All publicly available global sequences for the two endemic human coronaviruses were retrieved from NCBI GenBank (1983–2025). Recombination screening was performed on the partial spike alignments assembled for downstream analyses (HCoV-NL63: n = 295; HCoV-OC43: n = 262) using RDP5 [24]. Putative recombination signals were assessed using multiple algorithms implemented in RDP5 (RDP, GENECONV, BootScan, MaxChi, Chimaera, SiScan, 3Seq, and LARD). Recombination events were retained as putative candidates only when supported by at least five methods with P < 0.05. Because only partial spike sequences were analyzed, the results were interpreted as screening-level evidence, and recombination outside the sequenced region cannot be excluded.

### Time-resolved phylogenetics and migration dynamics

The final dataset comprised partial spike sequences of HCoV-NL63 (n = 295) and HCoV-OC43 (n = 259), combining newly generated Thai sequences with globally representative references retrieved from NCBI GenBank (1983–2025) (GenBank accession numbers for newly generated sequences are provided in S2 Table in S1 File). Sequences were aligned using MAFFT v7 [25], and partitioned by codon position (1st, 2nd, and 3rd) for downstream analyses. The best-fitting nucleotide substitution model for each dataset was determined by model testing using an information criterion with appropriate corrections. For the partial spike region, the Hasegawa–Kishino–Yano (HKY) model with gamma-distributed rate heterogeneity among sites and a proportion of invariant sites (HKY + G + I) was selected for HCoV-NL63, whereas the HKY model with gamma-distributed rate heterogeneity among sites (HKY + G) was selected for HCoV-OC43. Maximum-likelihood phylogenies were inferred in MEGA v10 [26], and node support was evaluated using 1,000 bootstrap replicates. Temporal signal was assessed in TempEST v1.5.3 by regressing root-to-tip genetic distance against sampling time [27].

Population dynamics were inferred in BEAST v1.10.4 [28] using an HKY substitution model with gamma-distributed among-site rate variation and an uncorrelated lognormal (UCLN) relaxed clock. Changes in effective population size through time were reconstructed under a Bayesian SkyGrid coalescent prior.

Spatial–temporal dissemination for each HCoV species was examined using a discrete-trait phylogeographic framework based on an asymmetric continuous-time Markov chain (CTMC) model. To mitigate sparse sampling in discrete-trait phylogeography, countries represented by few sequences were aggregated into broader geographic states. For example, the UK and France were grouped as Europe. State definitions were specified separately for each species based on data availability. For NL63, locations were grouped into Thailand, USA, China, Japan, Australia, Asia (other), Africa, Russia, and Europe. For HCoV-OC43, locations were grouped into Thailand, Japan, Australia, Canada, Asia (other), Africa, USA, Europe, and China. For both HCoV-NL63 and HCoV-OC43, “Asia (other)” included Asian countries excluding Thailand, China, and Japan, which were modeled as separate location states. These analyses were performed under a UCLN relaxed clock with an exponential-growth coalescent prior. MCMC chains were run for 300,000,000 generations, sampling every 30,000 steps. Convergence and sampling adequacy were evaluated in Tracer v1.7.1 [29], with effective sample sizes (ESS) exceeding 200 for key parameters after discarding a 20% burn-in. Posterior tree sets were summarized using TreeAnnotator v1.8.4 to generate maximum clade credibility (MCC) trees, and phylogenies were visualized in FigTree (https://github.com/rambaut/figtree/releases).

Support for individual diffusion pathways was quantified using Bayes factors (BF) from the BSSVS output, and lineage movement was summarized using Markov jump counts. Both BF summaries and Markov jump outputs were extracted from BEAST log files and processed in R software (version 4.4.2) [30], which was also used to generate all phylogeographic visualizations, including base maps constructed with the ‘rnaturalearth’ package (version 1.2.0) sourcing Natural Earth data. Diffusion pathways were classified by BF strength as follows: BF ≥ 1,000 (decisive), 100 ≤ BF < 1,000 (very strong), 10 ≤ BF < 100 (strong), and 3 ≤ BF < 10 (supported) [31].

### Amino-acid substitution enrichment analysis

Aligned spike protein sequences were grouped by sampling origin (Thailand vs non-Thailand) based on the dataset annotations in the FASTA headers. At each alignment position, amino-acid frequencies were counted separately for the two groups, and the reference residue was defined as the consensus (the most common amino acid across all sequences). For each non-reference residue (reference→alternate), differences in frequency between groups were evaluated with a two-sided Fisher’s exact test using a 2 × 2 table (alternate vs non-alternate by group). P-values were adjusted for multiple testing with the Benjamini–Hochberg method, and results were considered significant at FDR q < 0.05. We filtered out extremely infrequent substitutions by requiring at least three observations across all sequences (total alternate count ≥3). All analyses were performed in R (version 4.4.2) [30] using Biostrings, dplyr, and ggplot2/ggrepel. Enrichment is summarized by log2OR, with positive values indicating higher frequency in Thailand and negative values indicating higher frequency outside Thailand.

### Codon-based selection analyses (HyPhy)

To assess selection acting on the spike coding region, we performed codon-based analyses using complementary methods implemented in the HyPhy framework through the Datamonkey web server [32]. We applied Fixed Effects Likelihood (FEL), Mixed Effects Model of Evolution (MEME), and Fast Unconstrained Bayesian AppRoximation (FUBAR) [33–35]. These methods estimate site-specific nonsynonymous and synonymous substitution rates and test for evidence of purifying selection and episodic diversifying selection across codons, using the aligned nucleotide sequences and an associated phylogenetic tree as input. Sites were considered supported for diversifying selection if identified by FEL or MEME at p < 0.1, or by FUBAR with posterior probability > 0.90. Method-specific site lists and overlaps were summarized and reported.

## Results

### Epidemiology and molecular characterization of HCoV-NL63 and HCoV-OC43

**Fig 1:**
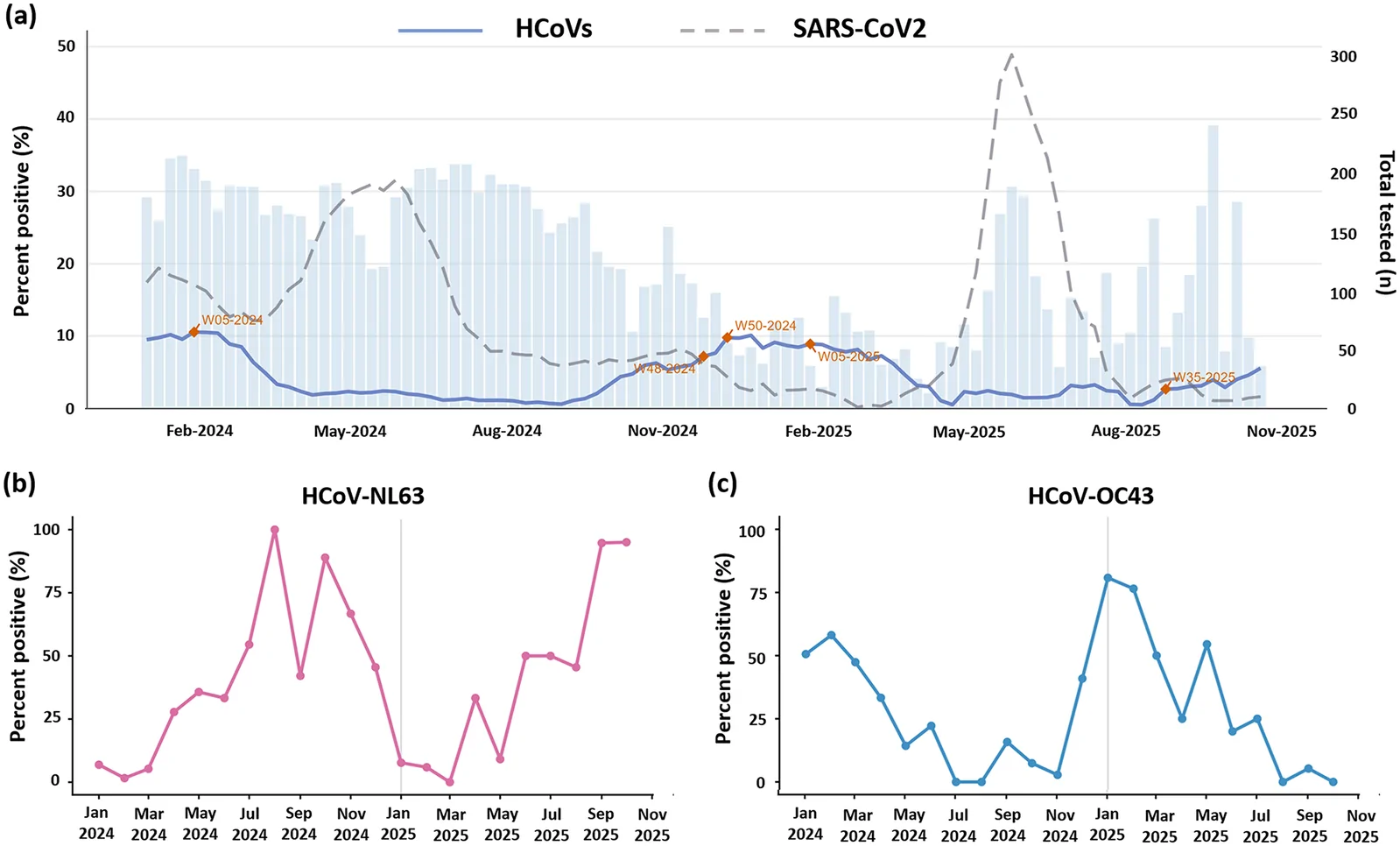
Weekly coronavirus positivity and species/genotype in Thailand, 2024–2025. (a) Weekly percent positivity shown as 5-week weighted moving average (WMA5) for endemic HCoVs (solid line) and SARS-CoV-2 (dashed line); bars indicate total ARI specimens tested (right axis). (b–c) Monthly genotype composition within HCoV-NL63 and HCoV-OC43, respectively.

During 2024–2025, we screened ARI specimens for common human coronaviruses (HCoVs) (Fig 1a). In 2024, 8,122 samples were tested and 329 were positive (4.05%), compared with 3,587 tested and 131 positive in 2025 (3.65%). Across the two years combined, 460 HCoV-positive samples were identified from 11,709 ARI hospitalizations screened (3.93%). By species, HCoV-OC43 was the most frequently detected (n = 163, 35.4% of positives), followed by HCoV-NL63 (n = 154, 33.5%), HCoV-HKU1 (n = 77, 16.7%), and HCoV-229E (n = 66, 14.3%). The 5-week weighted moving average (WMA5) for HCoVs showed a bimodal pattern in 2024. Activity increased early in the year, stayed low from roughly April through September, and then rose again in late November to December. In 2025, HCoV activity remained high in January to February but dropped to very low levels in March to April, followed by intermittent rebounds later in the year. SARS-CoV-2 showed distinct waves, including a marked surge in mid-2025. Weeks with high SARS-CoV-2 activity generally aligned with lower HCoV positivity, suggesting a temporal offset between endemic HCoVs and SARS-CoV-2 within the same weekly ARI testing stream.

Monthly species composition (Fig 1b–1c) suggested seasonal shifts in dominant lineages. In 2024, HCoV-OC43 accounted for the largest annual share (n = 115, 35.0% of 329 positives that year) and contributed a substantial proportion of detections early in the year, whereas HCoV-NL63 (n = 97, 29.5%) increased and predominated during the middle-to-late months; HCoV-HKU1 (n = 70, 21.3%) and HCoV-229E (n = 47, 14.3%) were detected at lower, fluctuating levels throughout the year (S2 Fig in S1 File). In 2025, total HCoV detections declined (n = 131); HCoV-NL63 became the most frequently detected species overall (n = 55, 42.0%), increasing in relative contribution during the second half of the year and aligning with the late-season spikes observed in the weekly series, while HCoV-OC43 remained substantial (n = 51, 38.9%) and dominant in the early months, consistent with the early-year wave. HCoV-229E (n = 19, 14.5%) and HCoV-HKU1 (n = 6, 4.6%) contributed smaller proportions in 2025, with monthly trends shown in S2 Fig in S1 File.

**Fig 2:**
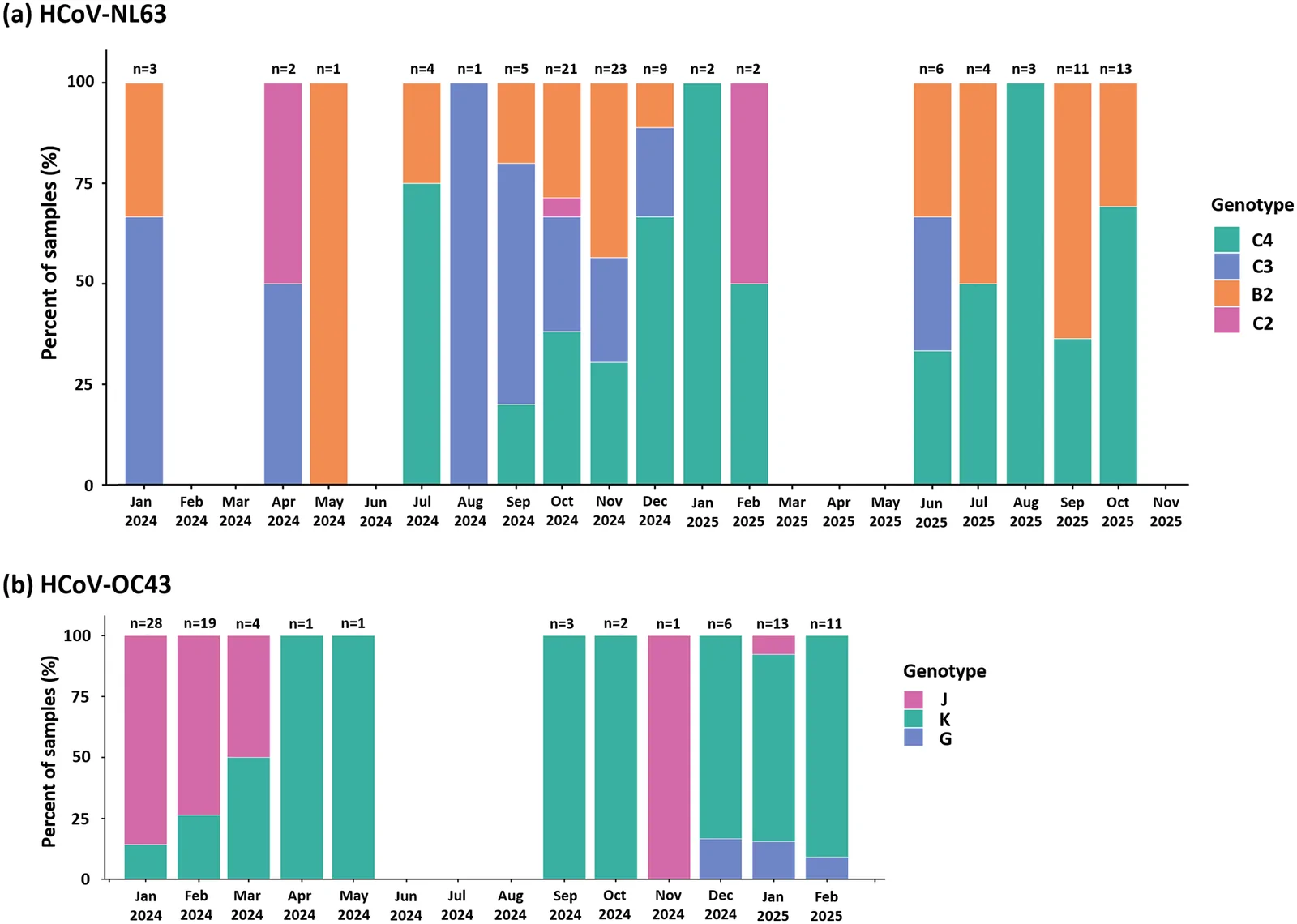
Monthly genotype composition of endemic human coronaviruses in Thailand, 2024–2025. (a) HCoV-NL63 and (b) HCoV-OC43. Stacked bars show the monthly proportion of sequences assigned to each genotype (percent of sequences per month). Numbers above bars indicate the number of sequences available for that month (n). Blank months indicate no sequences available for analysis (e.g., no detections and/or no sequencing performed).

Genotype assignments for HCoV-NL63 and HCoV-OC43 in this study were made relative to previously established, whole-genome-informed reference genotype classifications (HCoV-OC43, genotypes A–K [7]; HCoV-NL63, genotypes A–C [16,17]), rather than being newly defined from the partial spike sequences generated in this study. Thai sequences were placed into these reference genotype clades based on their phylogenetic position within maximum-likelihood trees constructed using the partial spike alignment together with global reference sequences representing each established genotype (S3 Fig in S1 File). Maximum-likelihood phylogenies supported genotype assignments with bootstrap support of at least 80 (S3 Fig in S1 File). Thai sequences did not form a single cluster. Instead, they were distributed among global reference sequences within each genotype, indicating the presence of multiple circulating lineages. During 2024–2025 in Thailand, both endemic coronaviruses showed clear genotype dominance patterns (Fig 2a–2b). For HCoV–OC43, the genotype distribution was largely bimodal, with K (43/89; 48.3%) and J (42/89; 47.2%) occurring at nearly equal frequencies, while G was infrequent (4/89; 4.5%). For HCoV–NL63, genotype C4 predominated (48/110; 43.6%), followed by B2 (36/110; 32.7%) and C3 (23/110; 20.9%), whereas C2 was rarely detected (3/110; 2.7%).

Recombination screening in RDP5 identified only a small number of putative signals in the partial spike alignments. Only one event in HCoV–NL63 and one event in HCoV–OC43 met the predefined support threshold (≥5 methods), and both involved non-Thai sequences sampled in France. Excluding these recombinant candidates did not alter the major clade structure of the maximum-likelihood phylogenies (S4–S5 Fig in S1 File). As this screening was restricted to the sequenced partial spike region, recombination breakpoints occurring elsewhere in the genome cannot be excluded. Root-to-tip regression further indicated a modest temporal signal (HCoV–NL63: R² = 0.242; HCoV–OC43: R² = 0.224; S6 Fig in S1 File).

### Evolutionary history and phylogeography of HCoV-NL63

**Fig 3:**
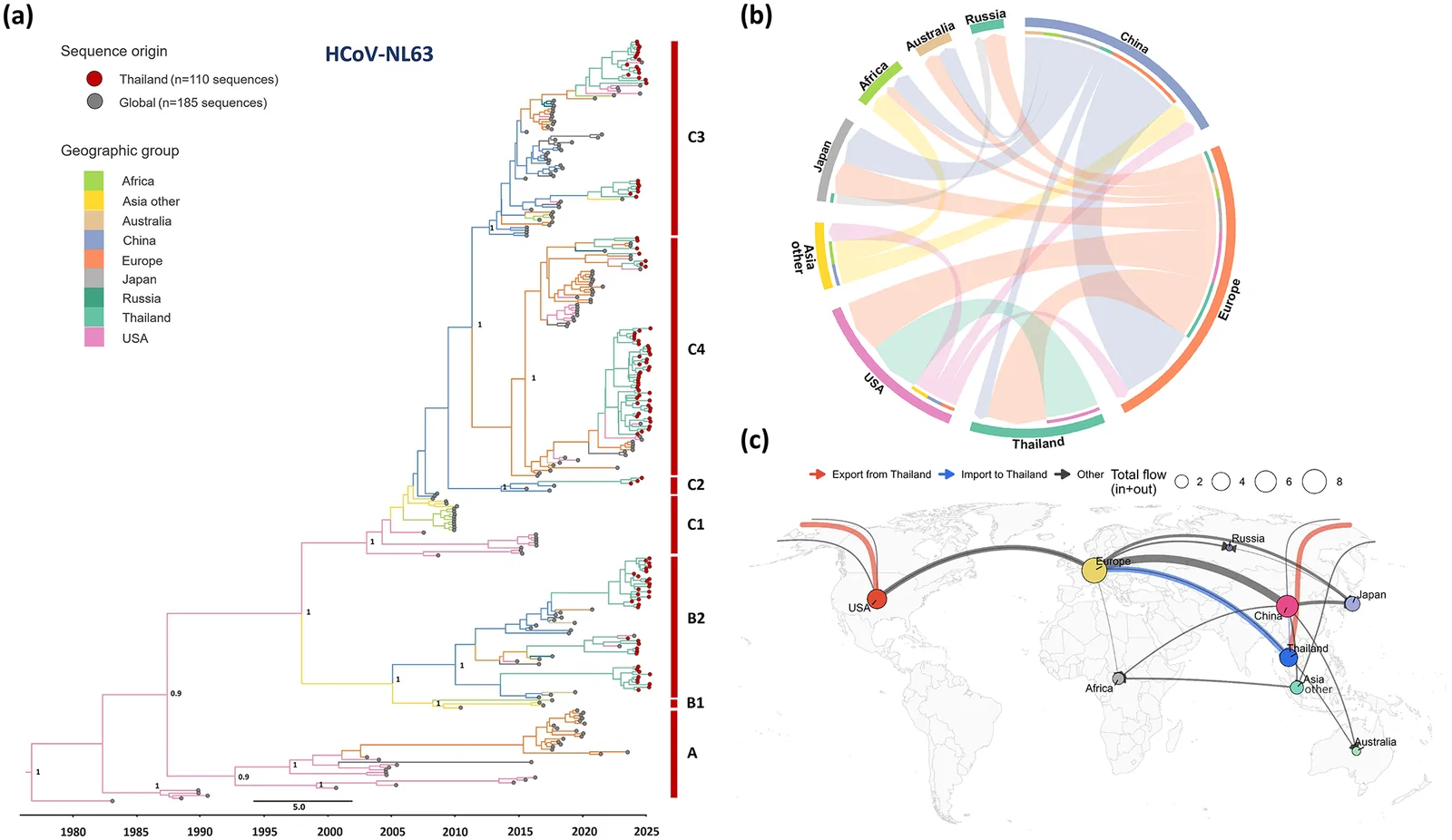
Bayesian phylogeography of HCoV-NL63 inferred by discrete-trait analysis (BSSVS). (a) Time-scaled maximum clade credibility (MCC) tree from BEAST; branches are coloured by the most probable geographic state, and tips indicate sequence origin (Thailand, red; global references, grey). Selected internal nodes are annotated with posterior probabilities. (b) Chord diagram summarising supported migration links (Bayes factor threshold as in Methods); ribbon width scales with inferred migration support/weight, and outer arcs indicate total connectivity per region. (c) Map of supported migration links; exports from Thailand are shown in red, imports into Thailand in blue, and other links in grey. Node size reflects total flow (in + out), and edge thickness scales with migration weight. The base map was generated using the R package ‘rnaturalearth’ (version 1.2.0; https://cran.r-project.org/package=rnaturalearth), which sources publicly available Natural Earth data.

The global evolutionary history of HCoV–NL63 was reconstructed from a time-scaled Bayesian phylogeny of partial spike sequences sampled worldwide to place the Thai sequences in a global context. The maximum clade credibility (MCC) tree recovered the expected genotype structure (A, B1–B2, and C1–C4), with strong support at the main backbone nodes (PP = 0.9–1.0; Fig 3a). Seven major clusters were resolved, comprising four clusters within genotype C, two within genotype B, and one within genotype A. Thai sequences (n = 110) did not form a single monophyletic clade. Instead, they were distributed across the tree and interspersed among global reference sequences (n = 185), consistent with multiple introductions into Thailand during 2024–2025 followed by local transmission in several subclusters.

The estimated substitution rate for HCoV–NL63 was 3.86 × 10^-4^ substitutions/site/year (95% HPD: 3.23 × 10^-4^–4.47 × 10^-4^). Within genotype B2, Thai sequences were separated into three subclusters. The largest Thai subcluster had a tMRCA of Aug 2022 (95% HPD: Dec 2020–Jan 2024). Within genotype C3, Thai sequences split into two subclusters, supporting more than one introduction during 2024–2025. The largest Thai subcluster dated to Jan 2022 (95% HPD: Sep 2020–Jun 2023). Within genotype C4, Thai sequences formed two subclusters, and the major Thai subcluster showed a tMRCA of May 2022 (95% HPD: Apr 2021–Jul 2023).

To investigate spatial diffusion, we used a discrete-trait phylogeographic model with nine geographic states, defined as a mix of individual countries and broader regional groupings based on sampling density. Using BSSVS, we summarized statistically supported diffusion links with Bayes factor (BF) > 3 (posterior indicator probability, p), identifying 18 supported migration routes (Fig 3b–3c). The strongest supported link connected China and Europe (BF = 31,994; p = 1.00), with additional major links involving Europe and East Asia/USA (Europe–USA: BF = 528; p = 0.99; China–Japan: BF = 248; p = 0.97; Europe–Japan: BF = 110; p = 0.93). Based on this partial-spike discrete-trait analysis, Thailand showed apparent bidirectional connectivity among supported links, with the most prominent inbound route from Europe (Europe–Thailand: BF = 62; p = 0.89) and the strongest outbound route to the USA (Thailand–USA: BF = 157; p = 0.95).

In discrete-trait phylogeographic reconstructions (nine geographic states), the posterior distribution supported a total of 13 introductions into Thailand (95% HPD interval: 10–17) (S3 Table in S1 File). Most inferred introductions originated from Europe (posterior mean = 9.56; 95% HPD: 0–15), with smaller contributions from China (mean = 1.21; 95% HPD: 0–5), the USA (mean = 1.03; 95% HPD: 0–8) and Japan (mean = 0.78; 95% HPD: 0–6), whereas other regions contributed fewer than one introduction on average and their HPD intervals included zero. Outbound transitions from Thailand were dominated by a single route to the USA (posterior mean = 8.14), while transitions to other regions were rare (posterior means ≤ ~0.5) and generally not consistently supported across trees.

### Evolutionary history and phylogeography of HCoV-OC43

**Fig 4:**
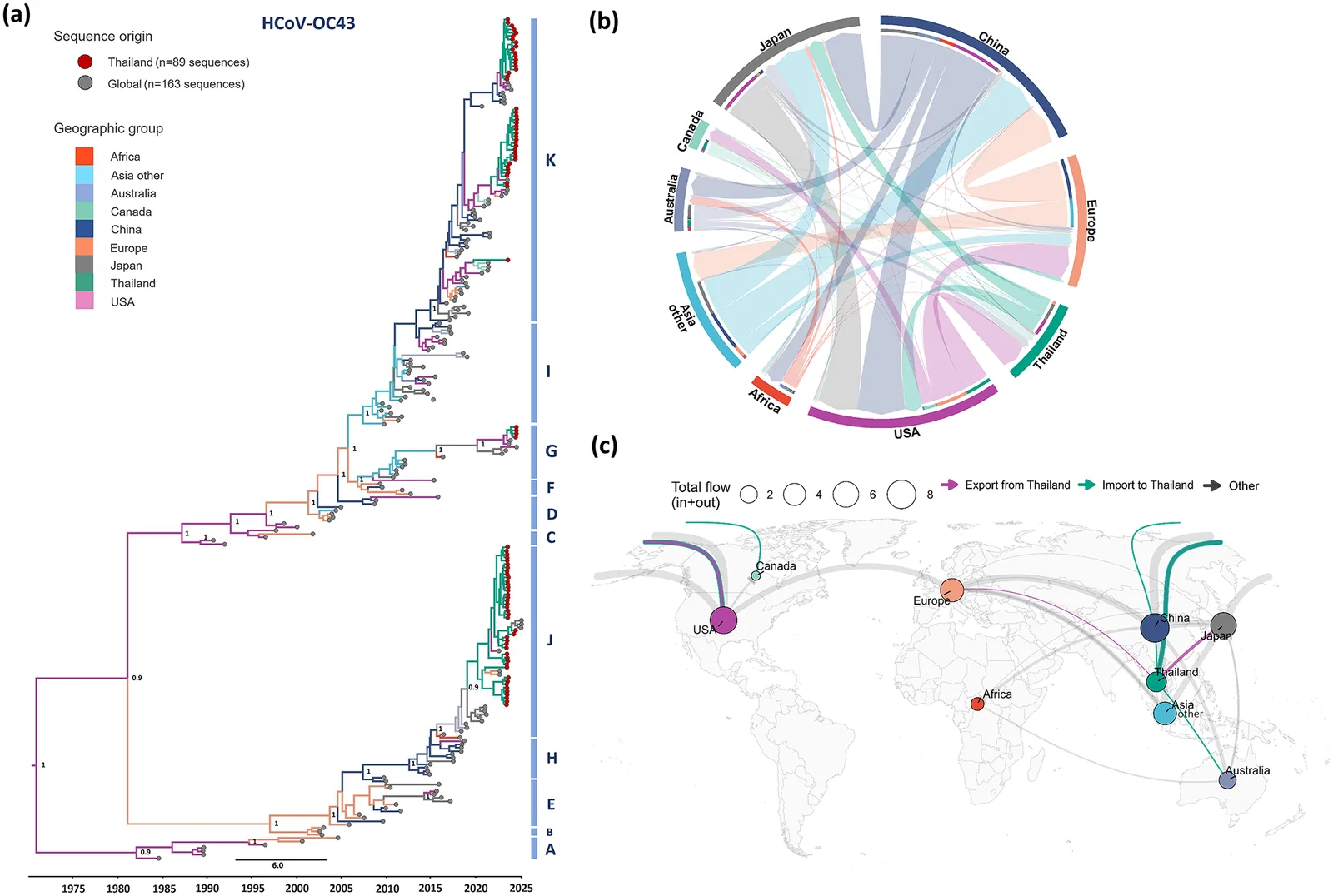
Bayesian phylogeography of HCoV-OC43 inferred by discrete-trait analysis (BSSVS). (a) Time-scaled maximum clade credibility (MCC) tree from BEAST; branches are coloured by the most probable geographic state, and tips indicate sequence origin (Thailand, red; global references, grey). Selected internal nodes are annotated with posterior probabilities. (b) Chord diagram summarising BSSVS-supported migration links (Bayes factor threshold as in Methods); ribbon width scales with inferred migration support/weight, and outer arcs indicate total connectivity per region. (c) Map of supported migration links; exports from Thailand are shown in red, imports into Thailand in blue, and other links in grey. Node size reflects total flow (in + out), and edge thickness scales with migration weight. The base map was generated using the R package ‘rnaturalearth’ (version 1.2.0; https://cran.r-project.org/package=rnaturalearth), which sources publicly available Natural Earth data.

As also observed for HCoV–NL63, Thai HCoV–OC43 sequences were dispersed across the phylogeny rather than forming a single dominant lineage. In the time-scaled MCC tree inferred from global partial spike sequences, Thai viruses (n = 89) mapped onto the established HCoV–OC43 genotype framework spanning A–K, supported by high posterior probabilities along the main backbone (PP = 0.9–1.0; Fig 4a). The scattering of Thai tips among global references (n = 163), together with the presence of multiple Thai subclusters, is most consistent with repeated introductions into Thailand followed by local onward transmission in more than one lineage, based on the partial spike topology; because HCoV-OC43 evolution is influenced by recombination, this inference may not fully reflect genome-wide transmission history.

The estimated substitution rate for HCoV-OC43 was 9.27 × 10^-4^ substitutions per site per year (95% HPD 7.83 × 10^-4^ to 1.07 × 10^-3^). Within genotype J, the main Thai associated cluster, which also included a small number of sequences from France in 2023, the USA in 2023, and Japan in 2025, had a tMRCA in Dec 2020 (95% HPD Nov 2019 to Dec 2021). In contrast, the Thai subcluster within genotype G was more recent with a tMRCA in Mar 2024 (95% HPD Dec 2023 to Jun 2024). Genotype K contained two Thai subclusters, with the larger cluster dating to Jul 2022 (95% HPD Jul 2021 to Mar 2023).

Migration routes for HCoV-OC43 were inferred under the same discrete trait framework and BF threshold used for HCoV-NL63. Using BSSVS with BF greater than 3, we identified 20 supported migration routes (Fig 4b and 4c). The inferred diffusion network, based on the sequenced partial spike region, showed broad transcontinental connectivity, with prominent links among the USA, China, and Europe. The strongest corridor connected the USA and Europe (BF 586.46; p 0.996), and additional high support links involved China and East Asia, including China to Japan (BF 197.07; p 0.987) and China to the USA (BF 167.51; p 0.985), as well as Europe to China (BF 149.40; p 0.983). Thailand was most clearly connected through an inbound link from the USA (BF 138.90; p 0.982) and an outbound link to Japan (BF 62.47; p 0.960). A Thailand to USA link was also supported but had much weaker support (BF 4.52; p 0.633), suggesting that the strongest Thailand associated corridors for HCoV-OC43 were not identical to those inferred for NL63.

Markov-jump summaries (post-burn-in) suggested that HCoV-OC43 movements involving Thailand were concentrated in links with the USA (S4 Table in S1 File). Overall, the posterior distribution implied a median of 6 introductions into Thailand (95% HPD 3–13) and 5 exports from Thailand (95% HPD 2–13). In contrast, transitions involving other regions were rare, with posterior medians of zero and HPD intervals including zero, consistent with sporadic transitions across the posterior.

### HCoV-NL63 and HCoV-OC43 population dynamics over time (bayesian skygrid)

**Fig 5:**
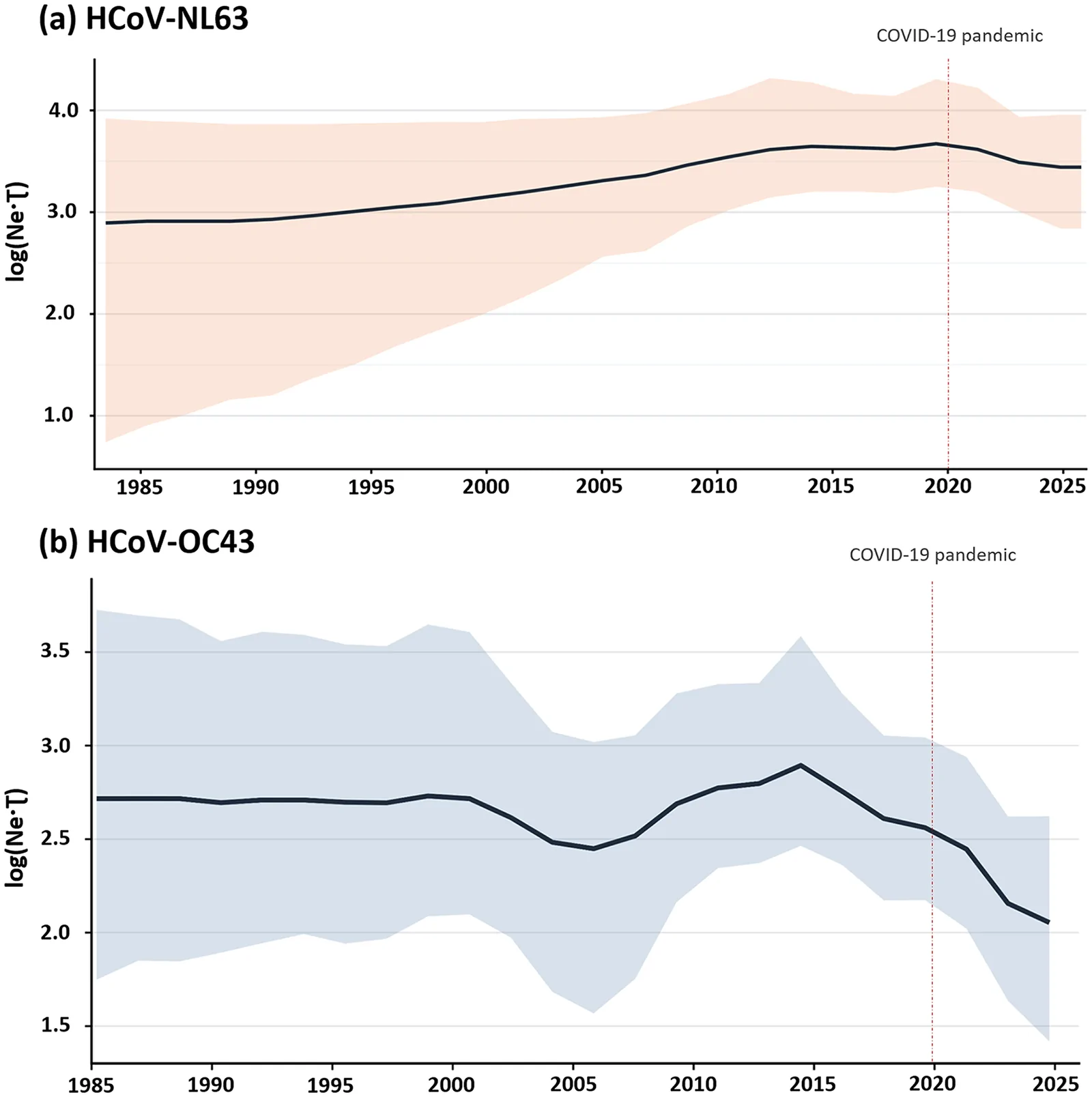
Bayesian Skygrid estimates of effective population size (Ne•τ) for (a.) OC43 and (b.) NL63. The black line represents the posterior median of log(Ne•τ), while the shaded area denotes the 95% highest posterior density (HPD) interval. The x-axis shows calendar time (years), anchored to the most recent sampling date so that the rightmost side corresponds to the present/end of the sampling window and the left side represents earlier time points. Higher log(Ne•τ) indicates larger effective population size (greater genetic diversity/ effective transmission), whereas lower values suggest contraction; uncertainty is reflected by the width of the HPD band.

To investigate long-term changes in viral genetic diversity through time, we inferred effective population size trajectories (log Ne·τ) for NL63 and OC43 using Bayesian Skygrid reconstructions (Fig 5). The two viruses showed contrasting demographic patterns. For NL63, the posterior median log(Ne·τ) rose toward the early 2000s and peaked around 2001 (median 3.317, 95% HPD 2.503–3.935), then gradually declined toward the most recent sampling date (14 Oct 2025; median 2.741, 95% HPD 0.164–3.868). OC43 displayed stronger temporal fluctuations, with a local minimum around 2003 (median 2.449, 95% HPD 1.568–3.019) followed by an increase to a peak near 2010 (median 2.731, 95% HPD 2.087–3.647). Credible intervals were widest in the earlier decades, consistent with sparser historical sampling and greater uncertainty deeper in time.

### Geographic enrichment of amino-acid substitutions and selection signals (TH vs non-TH)

**Fig 6:**
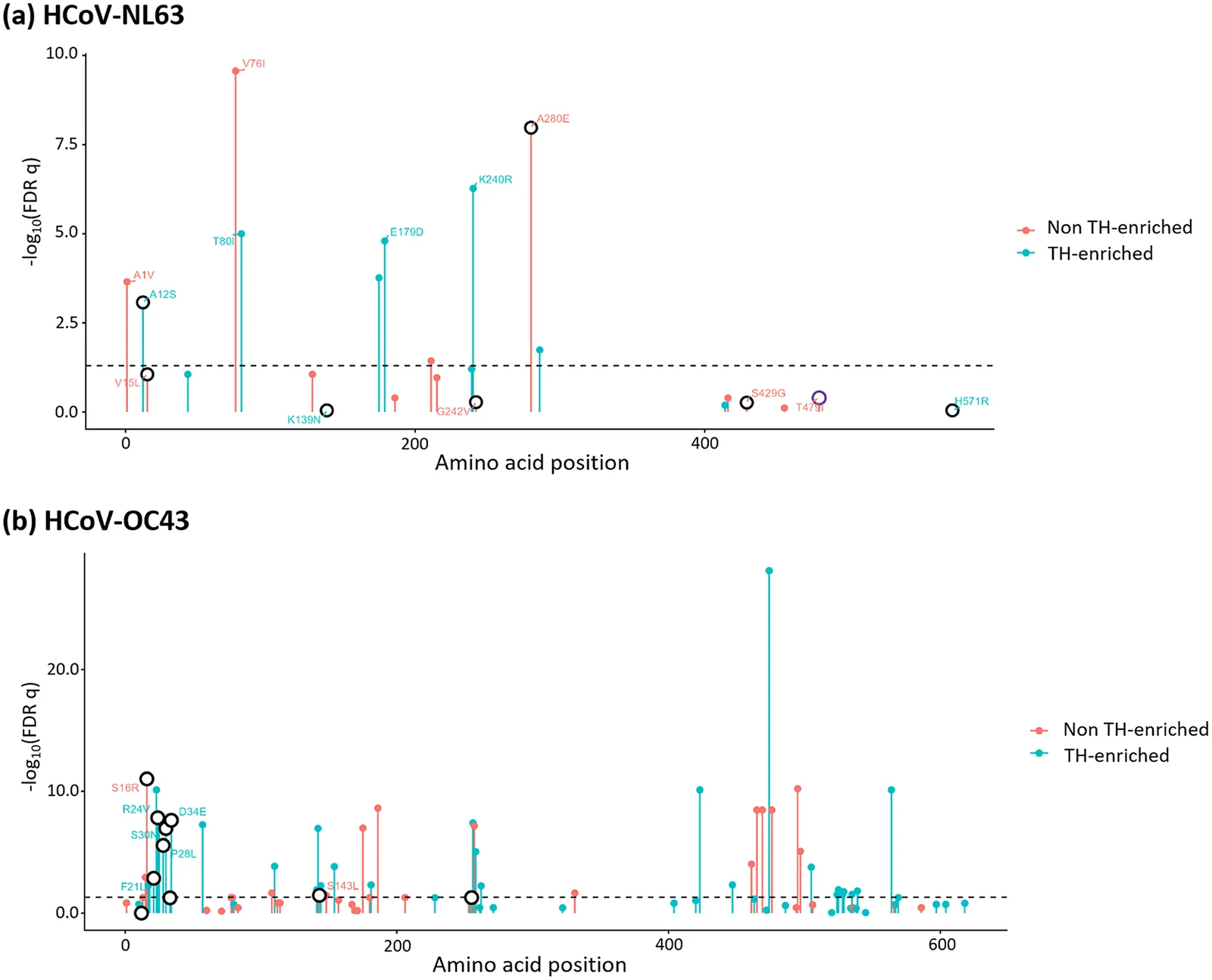
Enriched amino-acid substitutions in HCoV-NL63 and HCoV-OC43 (Thailand vs non-Thailand). Lollipop plots show amino-acid sites with differential substitution frequencies between Thai and non-Thai sequences for (a) HCoV-NL63 and (b) HCoV-OC43. For each alignment position, the most significant substitution is shown (one mutation per site). The y-axis reports −log10(FDR q) from Fisher’s exact tests with Benjamini–Hochberg correction. Colours indicate the direction of enrichment (Thailand-enriched vs non-Thailand–enriched), and the dashed line marks the significance threshold (FDR q = 0.05). Open circles denote codon sites supported by HyPhy selection analyses. In HCoV-OC43, circles highlight sites where enrichment overlaps codon-based evidence of diversifying selection (MEME and/or FEL/FUBAR), whereas in HCoV-NL63, circle outlines distinguish method-specific signals (FUBAR, grey outline; MEME, purple outline). Selected amino-acid changes are labeled.

To examine Thai–non-Thai differences in HCoV-OC43 and HCoV-NL63, amino-acid substitution enrichment was analyzed and compared with HyPhy selection analyses, with full substitution-level results (restricted to substitutions with total alternate count ≥ 3) provided in S5-S6 Table in S1 File. Comparison of Thai and non-Thai HCoV-NL63 sequences revealed multiple amino-acid positions with significant frequency differences (Fig 6a). Signals of positive selection were weak and not consistent across methods. FEL detected no sites, whereas FUBAR supported seven sites and MEME identified a single site at position 479, with no overlap between methods. Given this lack of concordance, the HCoV-NL63 results are presented mainly as enrichment patterns that may reflect lineage composition and sampling rather than a consensus set of positively selected sites. In HCoV-OC43, significant Thai–non-Thai differences were also observed at several amino-acid positions (Fig 6b). A subset of these substitutions overlapped codon sites supported for diversifying (positive) selection by HyPhy (MEME, together with FEL/FUBAR), including S16R, F21L, R24V, P28L, S30N, D34E, and S143L. Most of these overlap sites were Thailand-enriched, indicating higher frequencies in the Thai dataset. These overlapping sites were therefore prioritized as candidates for interpretation, while noting that frequency differences may still be influenced by lineage structure and sampling.

To assess whether Thailand–non-Thailand differences at key HCoV-OC43 sites were driven by genotype composition, genotype-stratified analyses were performed. Within genotype K, positions 16, 28, 30, and 34 remained strongly differentiated between Thai and non-Thai sequences, indicating that these patterns were not explained by genotype composition alone. Position 24 was largely linked to genotype J but still differed within J, whereas position 143 was rare in major genotypes and its apparent enrichment was driven by minor non-Thai genotypes (S7 Table in S1 File).

## Discussion

In this post–COVID-19 ARI surveillance study in Thailand, we show that HCoV-NL63 and HCoV-OC43 circulation during 2024–2025 was shaped by pronounced seasonality, dominance of a limited number of spike lineages with turnover, and repeated introductions within a partial-spike-inferred, structured diffusion network. By integrating routine detections with partial spike sequencing and time-scaled phylogenetic and phylogeographic analyses, we provide baseline genomic context for endemic HCoVs in a major Southeast Asian travel hub that remains underrepresented in public sequence data. Overall, the dispersion of Thai sequences across the global partial-spike phylogeny is consistent with a model of recurrent external seeding followed by local onward transmission across multiple co-circulating lineages, rather than long-term persistence of a single dominant lineage.

Weekly surveillance revealed clear seasonality and an apparent temporal offset between endemic HCoVs and SARS-CoV-2 within the same ARI testing stream. Endemic HCoV activity peaked during Thailand’s winter months, while SARS-CoV-2 surges tended to coincide with lower endemic HCoV positivity. This pattern is broadly consistent with winter-peaked endemic HCoV circulation reported in temperate settings, whereas tropical Southeast Asian studies describe more heterogeneous timing, including mid-year increases in HCoV-NL63 [9,36,37]. The offset from SARS-CoV-2 likely reflects a combination of post-pandemic shifts in healthcare-seeking and testing behavior and the effects of control measures and reopening that have been associated with virus-specific suppression and reemergence patterns in regional multiplex surveillance [38].

Both viruses showed marked temporal shifts in genotype composition, with strong seasonality and episodic dominance of specific genotypes. In the current study, we found no evidence of newly emerging genotypes in either HCoV-OC43 or HCoV-NL63, despite the established genotype diversity of HCoV-OC43 ranging from A to K [7] and HCoV-NL63 ranging from A to C [16,17]. For HCoV-OC43, the dominant genotype shifted between years. Genotype J was most common in 2024 and remained detectable at low frequency in 2025, whereas genotype K, detected in both years, became the main circulating genotype in 2025. This is consistent with a China study from 2017 to 2019 in which genotype K predominated [7]. However, the secondary genotype pattern differed. In that study, genotypes J and G were only sporadically detected, whereas in our dataset genotype J appeared repeatedly alongside genotype K and genotype G remained intermittent. In our dataset, HCoV-NL63 genotype C was dominant. By comparison, the China study reported genotype B as the major genotype [7]. Our findings indicate that endemic HCoVs experience continuous genotype turnover, with the predominant genotype varying by context, signifying alterations among circulating lineages rather than the emergence of new genotypes.

This study revealed that HCoV-OC43 showed a higher mean substitution rate than HCoV-NL63 (9.27 × 10^-4^ vs 3.86 × 10^-4^ substitutions/site/year), consistent with the stronger genotype turnover we observed for HCoV-OC43. When benchmarked against SARS-CoV-2 at approximately 6 × 10^-4^ substitutions per site per year, HCoV-OC43 appears to evolve faster, whereas HCoV-NL63 evolves more slowly [23,39]. The HCoV-NL63 rate is broadly consistent with values reported in some prior datasets from Amsterdam and China, while appearing lower than estimates reported in studies from Singapore and Tanzania during 2009–2018 [5,11,40]. By contrast, our HCoV-OC43 rate is closer to estimates reported from Singapore and Tanzania, while the Hong Kong study from 2004–2011 reported a lower rate than we observe here [11,13].

In this study, we found evidence for repeated introductions into Thailand for both HCoV-NL63 and HCoV-OC43, but the inferred corridors differed between viruses under the same discrete-trait framework. For HCoV-NL63, the strongest support pointed to introductions associated with Europe and an outbound link from Thailand to the USA. For HCoV-OC43, Thailand-associated links more clearly involved the USA and Japan, with weaker support for Thailand-to-USA. This virus-specific contrast is comparable to patterns reported in recent work from Singapore, which inferred more frequent long-range mixing and multi-region source dynamics for HCoV-OC43, while HCoV-NL63 showed less frequent international transmission and more sustained regional persistence [11]. Markov-jump summaries provided a relative measure of transition support across posterior trees, but they should not be interpreted as literal counts of importation events. Because discrete-trait inference depends on the global reference set, state aggregation, and uneven regional sampling, these corridors should be treated as context-dependent and may shift as Southeast Asian representation improves [41,42].

Our study showed that skygrid reconstructions from the global partial spike dataset reveal contrasting long-term demographic patterns for HCoV-NL63 and HCoV-OC43. HCoV-NL63 rose earlier, peaking around the early 2000s before declining toward the present, whereas HCoV-OC43 fluctuated more and peaked later around 2010. In the most recent years, both curves trend downward, and the drop is clearer for HCoV-OC43. This period falls within the COVID-19 era, when both transmission and everyday contact patterns shifted. At the same time, changes in healthcare use, testing practices, and how many sequences were generated could also shape what we see in the reconstruction. The broad shapes resemble prior reports, but direct quantitative comparisons are limited by differences in datasets and model settings [11]. Previous studies also showed that HCoV-OC43 genetic diversity during 2003–2012 was shaped by two transient bottlenecks around 2006 and 2008–2009, and suggested that genotype D contributed substantially to overall diversity during those years [43]. For HCoV-NL63, earlier skyline work reported a moderate rise in genetic diversity from 1989 to 2015, followed by a small decline near 2018 that was treated cautiously because recent sampling was limited [16].

Previous work on HCoV-OC43 and other seasonal HCoVs highlights spike as a key antigenic target and a common focus of evolutionary analyses [44,45]. Frequency-based comparisons are also widely used to contrast viral sequence variation between groups (e.g., across geography or lineages) [46–48]. We compared Thai and non-Thai amino-acid frequencies to identify geographically differentiated substitutions and then asked whether these sites overlap HyPhy-supported diversifying selection signals. Overall, enrichment mainly reflects geographic differentiation, with stronger HyPhy overlap in HCoV-OC43 than in HCoV-NL63 within the sequenced spike region, which for HCoV-OC43 predominantly covers the receptor-binding domain. This HCoV-OC43 pattern is broadly consistent with phylogenomic evidence from Singapore indicating continued global HCoV-OC43 turnover and intercontinental spread shaped by drift, recombination, and complex migration [11], although our selection signals cannot be generalized beyond the sequenced domain. We interpret overlap sites as candidates for follow-up (e.g., spike domain/epitope mapping and genotype-stratified checks), not as stand-alone proof of adaptation.

This study has several limitations. We analyzed partial spike sequences, which reduces resolution for fine-scale transmission inference and limits our ability to assess genome-wide processes, including recombination and selection. Whole-genome data would make lineage and genotype calls more reliable and would help us assess recombination more directly, especially for HCoV-OC43. The sequenced regions differed in their coverage of spike protein domains between the two species: the HCoV-NL63 partial sequence (1,875 nucleotides; 624 amino acids) covers the C-terminal portion of the S1 subunit, the S1/S2 cleavage region, and the N-terminal portion of the S2 subunit (including the fusion peptide), but does not include the receptor-binding domain (RBD); in contrast, the HCoV-OC43 partial sequence (1,842 nucleotides; 614 amino acids) encompasses the N-terminal domain and nearly the entirety of the RBD, including the receptor-binding motif, but does not extend into the S2 subunit. Because the RBD is subject to strong immune-driven diversifying selection and has been implicated as a recombination-prone region in other coronaviruses, genotype assignments and selection pressure analyses for HCoV-OC43 may be disproportionately influenced by evolutionary processes specific to this domain, whereas the HCoV-NL63 analysis, covering the more conserved S1/S2 and S2 fusion machinery, may better reflect genome-wide relationships but underrepresent RBD-driven diversification. Because tree topology and inferred migration routes are estimated from the sequenced locus, the same domain-specific constraints could also influence phylogeographic and migration inferences if recombination has decoupled the evolutionary history of the RBD from that of the rest of the genome, particularly for HCoV-OC43. These domain-specific differences should be considered when interpreting genotype assignments and phylogeographic inferences for each species, and genome-wide sequencing would be needed to fully resolve these limitations.

Phylogeographic results also depend on which global references are included and how locations are grouped into discrete states. Under-sampling in Southeast Asia and uneven temporal coverage elsewhere may bias inferred sources and sinks, overemphasize well-sampled regions, and widen uncertainty in BSSVS and Markov-jump summaries. Our sequence data were also drawn from hospital-based ARI surveillance and were not evenly available across months, which may affect apparent seasonality and may not fully reflect community transmission. Finally, limited clinical metadata prevented a more detailed assessment of lineage- or genotype-specific differences in disease severity.

In conclusion, our integrated surveillance and evolutionary analyses indicate that post–COVID-19 endemic HCoV circulation in Thailand during 2024–2025 was shaped by repeated introductions and regionally structured connectivity, with virus-specific differences in the dominant inferred corridors for HCoV-NL63 compared with HCoV-OC43. The seasonal patterns and genotype dominance reported here provide a baseline for Thailand and a reference point for interpreting future fluctuations or unusual clusters. Continued expansion of genomic surveillance for endemic respiratory viruses in Southeast Asia, supported by whole-genome sequencing and better regional representation, will strengthen phylogeographic inference, improve monitoring of lineage turnover, and clarify how endemic HCoVs co-circulate with other respiratory pathogens in the post-pandemic period.

## Supporting information

S1 File Supplementary information. This file contains Supplementary Figures 1–6 and Supplementary Tables 1–7.
(PDF)
