# Supplementary material for: Spatiotemporal dynamics and phylogeography of HCoV-NL63 and HCoV-OC43 in Thailand, 2024–2025
Source: PLoS One. 2026 Sep 8;21(9):e0357483. doi: 10.1371/journal.pone.0357483 (PMC13552785; doi:10.1371/journal.pone.0357483)
Supplement: Supplementary file 1 [file pone.0357483.s001.pdf]

Spatiotemporal dynamics and phylogeography of HCoV-NL63 and HCoV-OC43 in Thailand, 2024–2025

Jiratchaya Puenpa[1,2], Ratchadawan Aeemjinda[1], Preeyaporn Vichaiwattana[1], Sumeth Korkong[1], Yong Poovorawan[1,3*]

Supplementary Figure 1. Flow diagram of ARI hospitalizations screened for endemic HCoVs by real-time RT-PCR and the subset selected for HCoV-NL63 and HCoV-OC43 partial spike sequencing and downstream analyses.

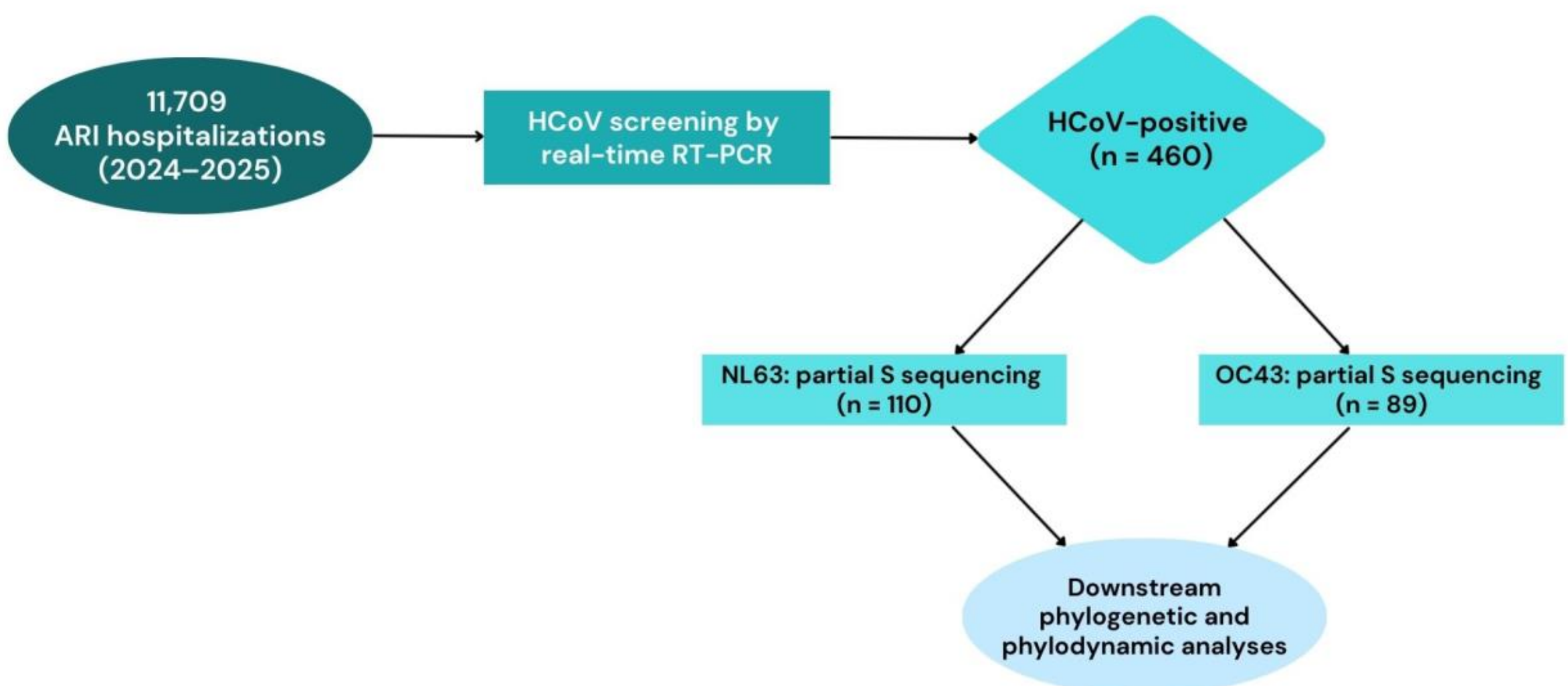

Supplementary Table 1. Primer sequences used for one-step RT-PCR amplification of partial spike (S) gene regions of HCoV-NL63 and HCoV-OC43.

Virus	No.	Primer	Sequence (5' to 3')
HCoV-NL63	1	NL63_F1	AAGAGTGGTAGGTTGTTRTTACG
		NL63_R1	ACTATAACACCACTCTTRAGATT
	2	NL63_F2	GCCACTGGTTCTGATGTTAATTG
		NL63_R2	ACTCACGAATACCAGAGACAGG
HCoV-OC43	1	OC43_F1	GGCTGCATGATGCTTAGACCAT
		OC43_R1	CAATTGGCTGAACAGTGTAACCG
	2	OC43_F2	ATGCTGTTGATTGTATGAGTGAT
		OC43_R2	CCTGAAATACCATARAGGTCAT

Supplementary Table 2. NCBI GenBank accession numbers for HCoV-NL63 and HCoV-OC43 partial spike (S) sequences generated in this study (2024–2025).

Virus	Isolate	Specimen type	Country	Collection date	Accession number
NL63	CU001	Nasopharyngeal swab	Thailand	20-Nov-24	PX987302
NL63	CU002	Nasopharyngeal swab	Thailand	21-Nov-24	PX987303
NL63	CU003	Nasopharyngeal swab	Thailand	21-Nov-24	PX987304
NL63	CU004	Nasopharyngeal swab	Thailand	21-Nov-24	PX987305
NL63	CU005	Nasopharyngeal swab	Thailand	24-Nov-24	PX987306
NL63	CU006	Nasopharyngeal swab	Thailand	24-Nov-24	PX987307
NL63	CU007	Nasopharyngeal swab	Thailand	25-Nov-24	PX987308
NL63	CU008	Nasopharyngeal swab	Thailand	25-Nov-24	PX987309
NL63	CU009	Nasopharyngeal swab	Thailand	26-Nov-24	PX987310
NL63	CU010	Nasopharyngeal swab	Thailand	27-Nov-24	PX987311
NL63	CU011	Nasopharyngeal swab	Thailand	27-Nov-24	PX987312
NL63	CU012	Nasopharyngeal swab	Thailand	1-Dec-24	PX987313
NL63	CU013	Nasopharyngeal swab	Thailand	2-Dec-24	PX987314
NL63	CU014	Nasopharyngeal swab	Thailand	5-Dec-24	PX987315
NL63	CU015	Nasopharyngeal swab	Thailand	5-Dec-24	PX987316
NL63	CU016	Nasopharyngeal swab	Thailand	6-Dec-24	PX987317
NL63	CU017	Nasopharyngeal swab	Thailand	6-Dec-24	PX987318
NL63	CU018	Nasopharyngeal swab	Thailand	11-Dec-24	PX987319
NL63	CU019	Nasopharyngeal swab	Thailand	13-Dec-24	PX987320
NL63	CU020	Nasopharyngeal swab	Thailand	16-Dec-24	PX987321
NL63	CU021	Nasopharyngeal swab	Thailand	4-Jan-25	PX987322
NL63	CU022	Nasopharyngeal swab	Thailand	2-Jun-25	PX987323

NL63	CU023	Nasopharyngeal swab	Thailand	2-Jul-25	PX987324
NL63	CU024	Nasopharyngeal swab	Thailand	8-Oct-24	PX987325
NL63	CU025	Nasopharyngeal swab	Thailand	9-Oct-24	PX987326
NL63	CU026	Nasopharyngeal swab	Thailand	12-Oct-24	PX987327
NL63	CU027	Nasopharyngeal swab	Thailand	12-Oct-24	PX987328
NL63	CU028	Nasopharyngeal swab	Thailand	13-Oct-24	PX987329
NL63	CU029	Nasopharyngeal swab	Thailand	16-Oct-24	PX987330
NL63	CU030	Nasopharyngeal swab	Thailand	16-Oct-24	PX987331
NL63	CU031	Nasopharyngeal swab	Thailand	23-Oct-24	PX987332
NL63	CU032	Nasopharyngeal swab	Thailand	24-Oct-24	PX987333
NL63	CU033	Nasopharyngeal swab	Thailand	26-Oct-24	PX987334
NL63	CU034	Nasopharyngeal swab	Thailand	26-Oct-24	PX987335
NL63	CU035	Nasopharyngeal swab	Thailand	26-Oct-24	PX987336
NL63	CU036	Nasopharyngeal swab	Thailand	29-Oct-24	PX987337
NL63	CU037	Nasopharyngeal swab	Thailand	30-Oct-24	PX987338
NL63	CU038	Nasopharyngeal swab	Thailand	1-Nov-24	PX987339
NL63	CU039	Nasopharyngeal swab	Thailand	6-Nov-24	PX987340
NL63	CU040	Nasopharyngeal swab	Thailand	7-Nov-24	PX987341
NL63	CU041	Nasopharyngeal swab	Thailand	9-Nov-24	PX987342
NL63	CU042	Nasopharyngeal swab	Thailand	10-Nov-24	PX987343
NL63	CU043	Nasopharyngeal swab	Thailand	11-Nov-24	PX987344
NL63	CU044	Nasopharyngeal swab	Thailand	15-Nov-24	PX987345
NL63	CU045	Nasopharyngeal swab	Thailand	16-Nov-24	PX987346
NL63	CU046	Nasopharyngeal swab	Thailand	17-Nov-24	PX987347
NL63	CU047	Nasopharyngeal swab	Thailand	18-Nov-24	PX987348
NL63	CU048	Nasopharyngeal swab	Thailand	20-Aug-24	PX987349
NL63	CU049	Nasopharyngeal swab	Thailand	22-Sep-24	PX987350
NL63	CU050	Nasopharyngeal swab	Thailand	24-Sep-24	PX987351
NL63	CU051	Nasopharyngeal swab	Thailand	26-Sep-24	PX987352
NL63	CU052	Nasopharyngeal swab	Thailand	29-Sep-24	PX987353
NL63	CU053	Nasopharyngeal swab	Thailand	3-Oct-24	PX987354
NL63	CU054	Nasopharyngeal swab	Thailand	3-Oct-24	PX987355
NL63	CU055	Nasopharyngeal swab	Thailand	5-Oct-24	PX987356
NL63	CU056	Nasopharyngeal swab	Thailand	6-Oct-24	PX987357
NL63	CU057	Nasopharyngeal swab	Thailand	7-Oct-24	PX987358
NL63	CU058	Nasopharyngeal swab	Thailand	8-Oct-24	PX987359
NL63	CU059	Nasopharyngeal swab	Thailand	3-Jan-24	PX987360
NL63	CU060	Nasopharyngeal swab	Thailand	10-Jan-24	PX987361
NL63	CU061	Nasopharyngeal swab	Thailand	15-Jan-24	PX987362
NL63	CU062	Nasopharyngeal swab	Thailand	14-Apr-24	PX987363
NL63	CU063	Nasopharyngeal swab	Thailand	15-Apr-24	PX987364

NL63	CU064	Nasopharyngeal swab	Thailand	18-May-24	PX987365
NL63	CU065	Nasopharyngeal swab	Thailand	4-Jul-24	PX987366
NL63	CU066	Nasopharyngeal swab	Thailand	8-Jul-24	PX987367
NL63	CU067	Nasopharyngeal swab	Thailand	10-Jul-24	PX987368
NL63	CU068	Nasopharyngeal swab	Thailand	22-Jul-24	PX987369
NL63	CU069	Nasopharyngeal swab	Thailand	22-Sep-24	PX987370
NL63	CU070	Nasopharyngeal swab	Thailand	15-Oct-24	PX987371
NL63	CU071	Nasopharyngeal swab	Thailand	10-Nov-24	PX987372
NL63	CU072	Nasopharyngeal swab	Thailand	10-Nov-24	PX987373
NL63	CU073	Nasopharyngeal swab	Thailand	25-Jan-25	PX987374
NL63	CU074	Nasopharyngeal swab	Thailand	2-Jun-25	PX987375
NL63	CU075	Nasopharyngeal swab	Thailand	2-Jul-25	PX987376
NL63	CU076	Nasopharyngeal swab	Thailand	14-Feb-25	PX987377
NL63	CU077	Nasopharyngeal swab	Thailand	20-Feb-25	PX987378
NL63	CU078	Nasopharyngeal swab	Thailand	1-Jun-25	PX987379
NL63	CU079	Nasopharyngeal swab	Thailand	17-Jun-25	PX987380
NL63	CU080	Nasopharyngeal swab	Thailand	19-Jun-25	PX987381
NL63	CU081	Nasopharyngeal swab	Thailand	24-Jun-25	PX987382
NL63	CU082	Nasopharyngeal swab	Thailand	5-Jul-25	PX987383
NL63	CU083	Nasopharyngeal swab	Thailand	30-Jul-25	PX987384
NL63	CU084	Nasopharyngeal swab	Thailand	6-Oct-25	PX987385
NL63	CU085	Nasopharyngeal swab	Thailand	7-Oct-25	PX987386
NL63	CU086	Nasopharyngeal swab	Thailand	7-Oct-25	PX987387
NL63	CU087	Nasopharyngeal swab	Thailand	7-Oct-25	PX987388
NL63	CU088	Nasopharyngeal swab	Thailand	10-Oct-25	PX987389
NL63	CU089	Nasopharyngeal swab	Thailand	14-Oct-25	PX987390
NL63	CU090	Nasopharyngeal swab	Thailand	14-Oct-25	PX987391
NL63	CU091	Nasopharyngeal swab	Thailand	6-Oct-25	PX987392
NL63	CU092	Nasopharyngeal swab	Thailand	17-Aug-25	PX987393
NL63	CU093	Nasopharyngeal swab	Thailand	20-Aug-25	PX987394
NL63	CU094	Nasopharyngeal swab	Thailand	28-Aug-25	PX987395
NL63	CU095	Nasopharyngeal swab	Thailand	11-Sep-25	PX987396
NL63	CU096	Nasopharyngeal swab	Thailand	13-Sep-25	PX987397
NL63	CU097	Nasopharyngeal swab	Thailand	23-Sep-25	PX987398
NL63	CU098	Nasopharyngeal swab	Thailand	24-Sep-25	PX987399
NL63	CU099	Nasopharyngeal swab	Thailand	24-Sep-25	PX987400
NL63	CU100	Nasopharyngeal swab	Thailand	24-Sep-25	PX987401
NL63	CU101	Nasopharyngeal swab	Thailand	24-Sep-25	PX987402
NL63	CU102	Nasopharyngeal swab	Thailand	12-Sep-25	PX987403
NL63	CU103	Nasopharyngeal swab	Thailand	21-Sep-25	PX987404
NL63	CU104	Nasopharyngeal swab	Thailand	24-Sep-25	PX987405

NL63	CU105	Nasopharyngeal swab	Thailand	24-Sep-25	PX987406
NL63	CU106	Nasopharyngeal swab	Thailand	8-Oct-25	PX987407
NL63	CU107	Nasopharyngeal swab	Thailand	9-Oct-25	PX987408
NL63	CU108	Nasopharyngeal swab	Thailand	10-Oct-25	PX987409
NL63	CU109	Nasopharyngeal swab	Thailand	10-Oct-25	PX987410
NL63	CU110	Nasopharyngeal swab	Thailand	10-Oct-25	PX987411
OC43	CU001	Nasopharyngeal swab	Thailand	6-Jan-24	PX987412
OC43	CU002	Nasopharyngeal swab	Thailand	8-Jan-24	PX987413
OC43	CU003	Nasopharyngeal swab	Thailand	12-Jan-24	PX987414
OC43	CU004	Nasopharyngeal swab	Thailand	12-Jan-24	PX987415
OC43	CU005	Nasopharyngeal swab	Thailand	15-Jan-24	PX987416
OC43	CU006	Nasopharyngeal swab	Thailand	20-Jan-24	PX987417
OC43	CU007	Nasopharyngeal swab	Thailand	26-Jan-24	PX987418
OC43	CU008	Nasopharyngeal swab	Thailand	3-Feb-24	PX987419
OC43	CU009	Nasopharyngeal swab	Thailand	7-Feb-24	PX987420
OC43	CU010	Nasopharyngeal swab	Thailand	13-Feb-24	PX987421
OC43	CU011	Nasopharyngeal swab	Thailand	13-Feb-24	PX987422
OC43	CU012	Nasopharyngeal swab	Thailand	14-Feb-24	PX987423
OC43	CU013	Nasopharyngeal swab	Thailand	14-Feb-24	PX987424
OC43	CU014	Nasopharyngeal swab	Thailand	20-Feb-24	PX987425
OC43	CU015	Nasopharyngeal swab	Thailand	3-Mar-24	PX987426
OC43	CU016	Nasopharyngeal swab	Thailand	5-Mar-24	PX987427
OC43	CU017	Nasopharyngeal swab	Thailand	28-Feb-24	PX987428
OC43	CU018	Nasopharyngeal swab	Thailand	14-May-24	PX987429
OC43	CU019	Nasopharyngeal swab	Thailand	18-Sep-24	PX987430
OC43	CU020	Nasopharyngeal swab	Thailand	31-Oct-24	PX987431
OC43	CU021	Nasopharyngeal swab	Thailand	16-Dec-24	PX987432
OC43	CU022	Nasopharyngeal swab	Thailand	5-Jan-24	PX987433
OC43	CU023	Nasopharyngeal swab	Thailand	7-Jan-24	PX987434
OC43	CU024	Nasopharyngeal swab	Thailand	8-Jan-24	PX987435
OC43	CU025	Nasopharyngeal swab	Thailand	9-Jan-24	PX987436
OC43	CU026	Nasopharyngeal swab	Thailand	12-Jan-24	PX987437
OC43	CU027	Nasopharyngeal swab	Thailand	14-Jan-24	PX987438
OC43	CU028	Nasopharyngeal swab	Thailand	16-Jan-24	PX987439
OC43	CU029	Nasopharyngeal swab	Thailand	19-Jan-24	PX987440
OC43	CU030	Nasopharyngeal swab	Thailand	20-Jan-24	PX987441
OC43	CU031	Nasopharyngeal swab	Thailand	23-Jan-24	PX987442
OC43	CU032	Nasopharyngeal swab	Thailand	25-Jan-24	PX987443
OC43	CU033	Nasopharyngeal swab	Thailand	26-Jan-24	PX987444
OC43	CU034	Nasopharyngeal swab	Thailand	28-Jan-24	PX987445
OC43	CU035	Nasopharyngeal swab	Thailand	28-Jan-24	PX987446

OC43	CU036	Nasopharyngeal swab	Thailand	30-Jan-24	PX987447
OC43	CU037	Nasopharyngeal swab	Thailand	30-Jan-24	PX987448
OC43	CU038	Nasopharyngeal swab	Thailand	1-Feb-24	PX987449
OC43	CU039	Nasopharyngeal swab	Thailand	1-Feb-24	PX987450
OC43	CU040	Nasopharyngeal swab	Thailand	3-Feb-24	PX987451
OC43	CU041	Nasopharyngeal swab	Thailand	4-Feb-24	PX987452
OC43	CU042	Nasopharyngeal swab	Thailand	5-Feb-24	PX987453
OC43	CU043	Nasopharyngeal swab	Thailand	9-Feb-24	PX987454
OC43	CU044	Nasopharyngeal swab	Thailand	13-Feb-24	PX987455
OC43	CU045	Nasopharyngeal swab	Thailand	16-Feb-24	PX987456
OC43	CU046	Nasopharyngeal swab	Thailand	21-Feb-24	PX987457
OC43	CU047	Nasopharyngeal swab	Thailand	26-Feb-24	PX987458
OC43	CU048	Nasopharyngeal swab	Thailand	3-Mar-24	PX987459
OC43	CU049	Nasopharyngeal swab	Thailand	14-Mar-24	PX987460
OC43	CU050	Nasopharyngeal swab	Thailand	1-Apr-24	PX987461
OC43	CU051	Nasopharyngeal swab	Thailand	13-Sep-24	PX987462
OC43	CU052	Nasopharyngeal swab	Thailand	24-Sep-24	PX987463
OC43	CU053	Nasopharyngeal swab	Thailand	25-Oct-24	PX987464
OC43	CU054	Nasopharyngeal swab	Thailand	3-Nov-24	PX987465
OC43	CU055	Nasopharyngeal swab	Thailand	11-Dec-24	PX987466
OC43	CU056	Nasopharyngeal swab	Thailand	13-Dec-24	PX987467
OC43	CU057	Nasopharyngeal swab	Thailand	17-Dec-24	PX987468
OC43	CU058	Nasopharyngeal swab	Thailand	23-Dec-24	PX987469
OC43	CU059	Nasopharyngeal swab	Thailand	1-Jan-24	PX987470
OC43	CU060	Nasopharyngeal swab	Thailand	1-Jan-24	PX987471
OC43	CU061	Nasopharyngeal swab	Thailand	6-Jan-24	PX987472
OC43	CU062	Nasopharyngeal swab	Thailand	16-Jan-24	PX987473
OC43	CU063	Nasopharyngeal swab	Thailand	26-Jan-24	PX987474
OC43	CU064	Nasopharyngeal swab	Thailand	27-Feb-24	PX987475
OC43	CU065	Nasopharyngeal swab	Thailand	24-Dec-24	PX987476
OC43	CU066	Nasopharyngeal swab	Thailand	10-Jan-25	PX987477
OC43	CU067	Nasopharyngeal swab	Thailand	8-Jan-25	PX987478
OC43	CU068	Nasopharyngeal swab	Thailand	8-Jan-25	PX987479
OC43	CU069	Nasopharyngeal swab	Thailand	15-Jan-25	PX987480
OC43	CU070	Nasopharyngeal swab	Thailand	16-Jan-25	PX987481
OC43	CU071	Nasopharyngeal swab	Thailand	17-Jan-25	PX987482
OC43	CU072	Nasopharyngeal swab	Thailand	18-Jan-25	PX987483
OC43	CU073	Nasopharyngeal swab	Thailand	18-Jan-25	PX987484
OC43	CU074	Nasopharyngeal swab	Thailand	22-Jan-25	PX987485
OC43	CU075	Nasopharyngeal swab	Thailand	22-Jan-25	PX987486
OC43	CU076	Nasopharyngeal swab	Thailand	27-Jan-25	PX987487

OC43	CU077	Nasopharyngeal swab	Thailand	29-Jan-25	PX987488
OC43	CU078	Nasopharyngeal swab	Thailand	29-Jan-25	PX987489
OC43	CU079	Nasopharyngeal swab	Thailand	5-Feb-25	PX987490
OC43	CU080	Nasopharyngeal swab	Thailand	5-Feb-25	PX987491
OC43	CU081	Nasopharyngeal swab	Thailand	6-Feb-25	PX987492
OC43	CU082	Nasopharyngeal swab	Thailand	6-Feb-25	PX987493
OC43	CU083	Nasopharyngeal swab	Thailand	7-Feb-25	PX987494
OC43	CU084	Nasopharyngeal swab	Thailand	7-Feb-25	PX987495
OC43	CU085	Nasopharyngeal swab	Thailand	9-Feb-25	PX987496
OC43	CU086	Nasopharyngeal swab	Thailand	10-Feb-25	PX987497
OC43	CU087	Nasopharyngeal swab	Thailand	11-Feb-25	PX987498
OC43	CU088	Nasopharyngeal swab	Thailand	12-Feb-25	PX987499
OC43	CU089	Nasopharyngeal swab	Thailand	13-Feb-25	PX987500

Supplementary Figure 2. Monthly genotype composition among coronavirus-positive samples for (a) HCoV-HKU1 and (b) HCoV-229E, respectively.

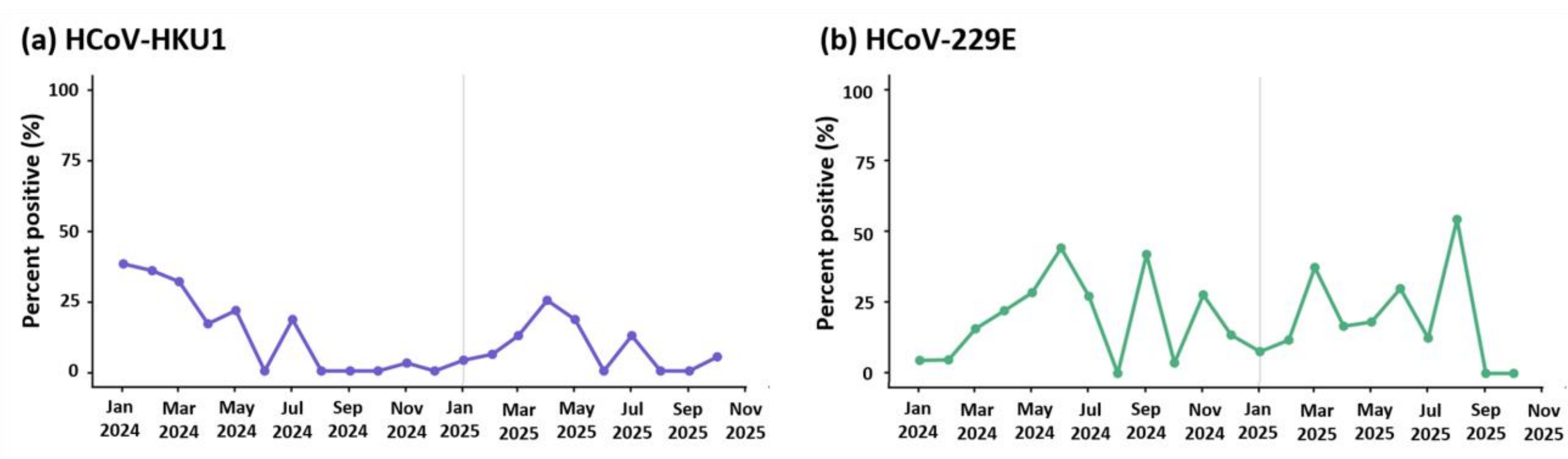

Supplementary Figure 3. Maximum-likelihood phylogenies of endemic human coronaviruses in Thailand (2024–2025). Maximum-likelihood (ML) trees inferred from partial spike (S) gene sequences of (a) HCoV-NL63 and (b) HCoV-OC43 sampled from hospitalized acute respiratory infection (ARI) patients in Thailand, during 2024–2025, together with representative global reference sequences spanning established genotypes. Thai sequences are highlighted in red (2024) and blue (2025). Vertical bars denote major genotype clades. Node support is shown as ML bootstrap values for selected internal nodes (≥80%; 1,000 replicates). Scale bars indicate nucleotide substitutions per site.

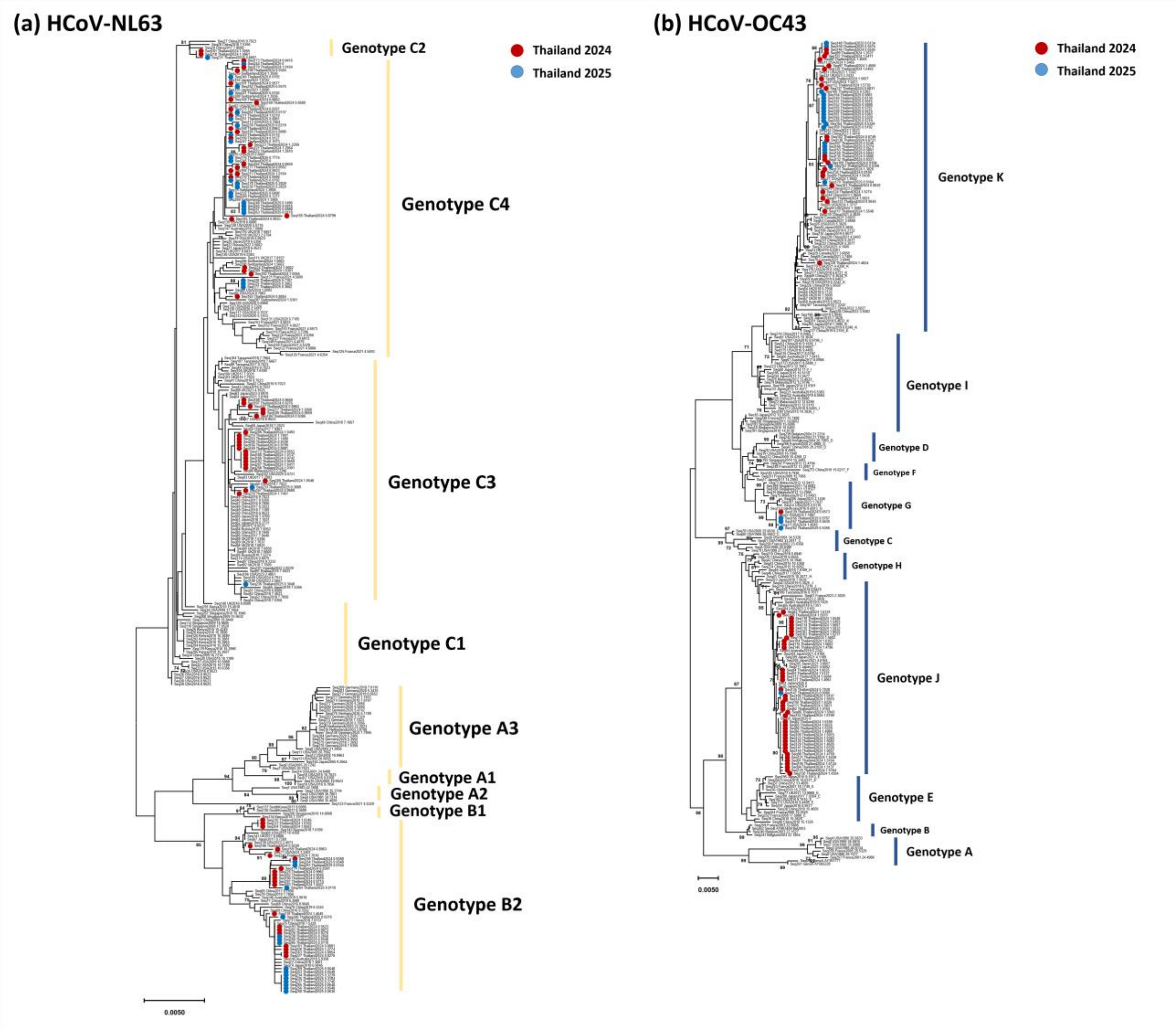

(a) HCoV-NL63
Thailand 2024
Thailand 2025
Genotype C2
Genotype C4
Genotype C3
Genotype C1
Genotype A3
Genotype A1
Genotype A2
Genotype B1
Genotype B2
0.0050
(b) HCoV-OC43
Thailand 2024
Thailand 2025
Genotype K
Genotype I
Genotype D
Genotype F
Genotype G
Genotype C
Genotype H
Genotype J
Genotype E
Genotype B
Genotype A
0.0050

Supplementary Figure 4. Effect of excluding a putative recombinant on the HCoV-NL63 phylogeny. Maximum-likelihood (ML) trees inferred from partial spike (S) gene sequences are shown (a) before and (b) after removal of one putative recombinant sequence identified by RDP5. Thai sequences are highlighted by sampling year (2024, red; 2025, blue). In panel (a), the putative recombinant is labeled in red and indicated by a red star. Scale bars indicate nucleotide substitutions per site.

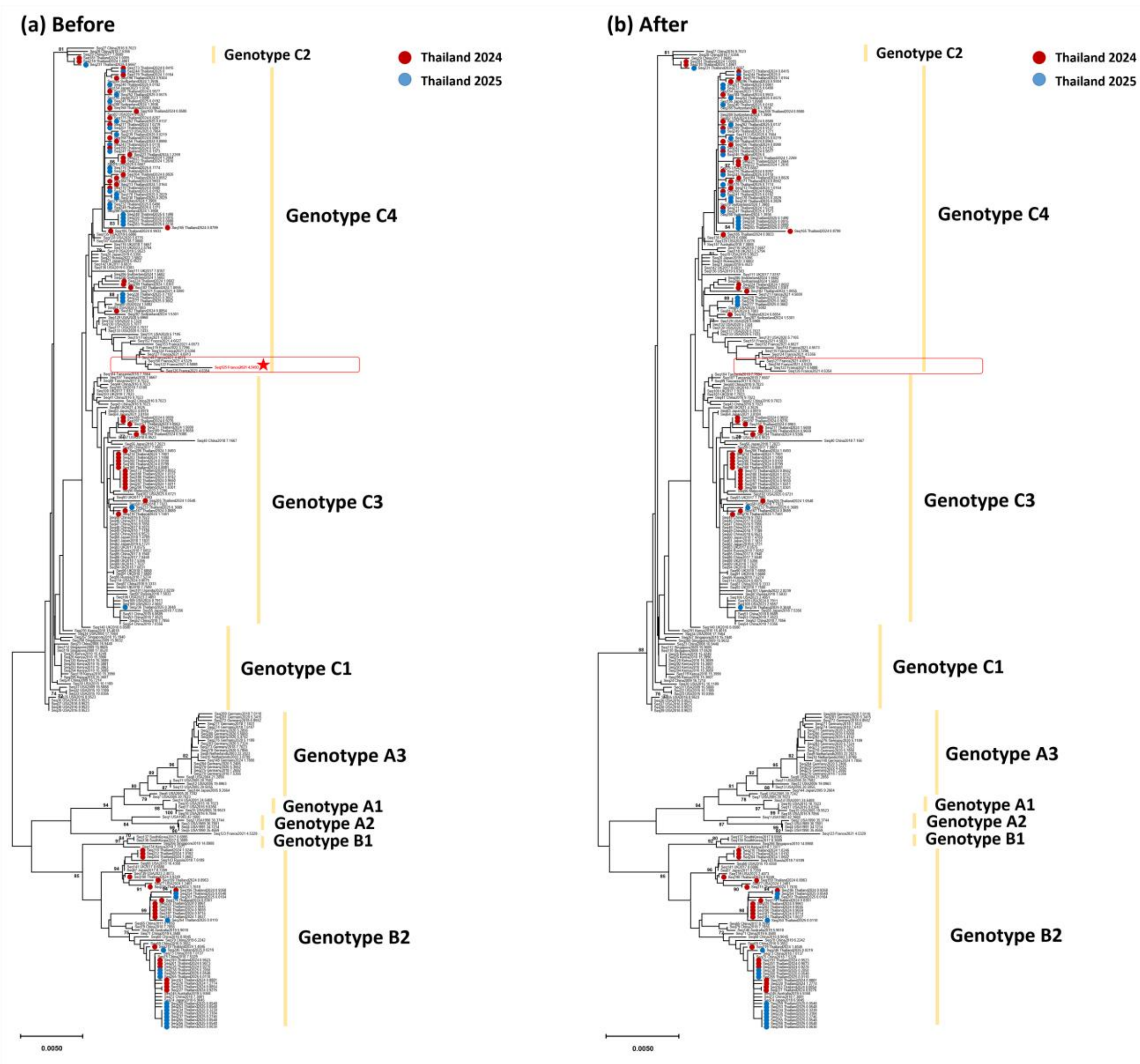

Supplementary Figure 5. Effect of excluding a putative recombinant on the HCoV-OC43 phylogeny. Maximum-likelihood (ML) trees inferred from partial spike (S) gene sequences are shown (a) before and (b) after removal of one putative recombinant sequence identified by RDP5. Thai sequences are highlighted by sampling year (2024, red circles; 2025, blue circles). In panel (a), the putative recombinant is labeled in red and indicated by a red star. Scale bars indicate nucleotide substitutions per site.

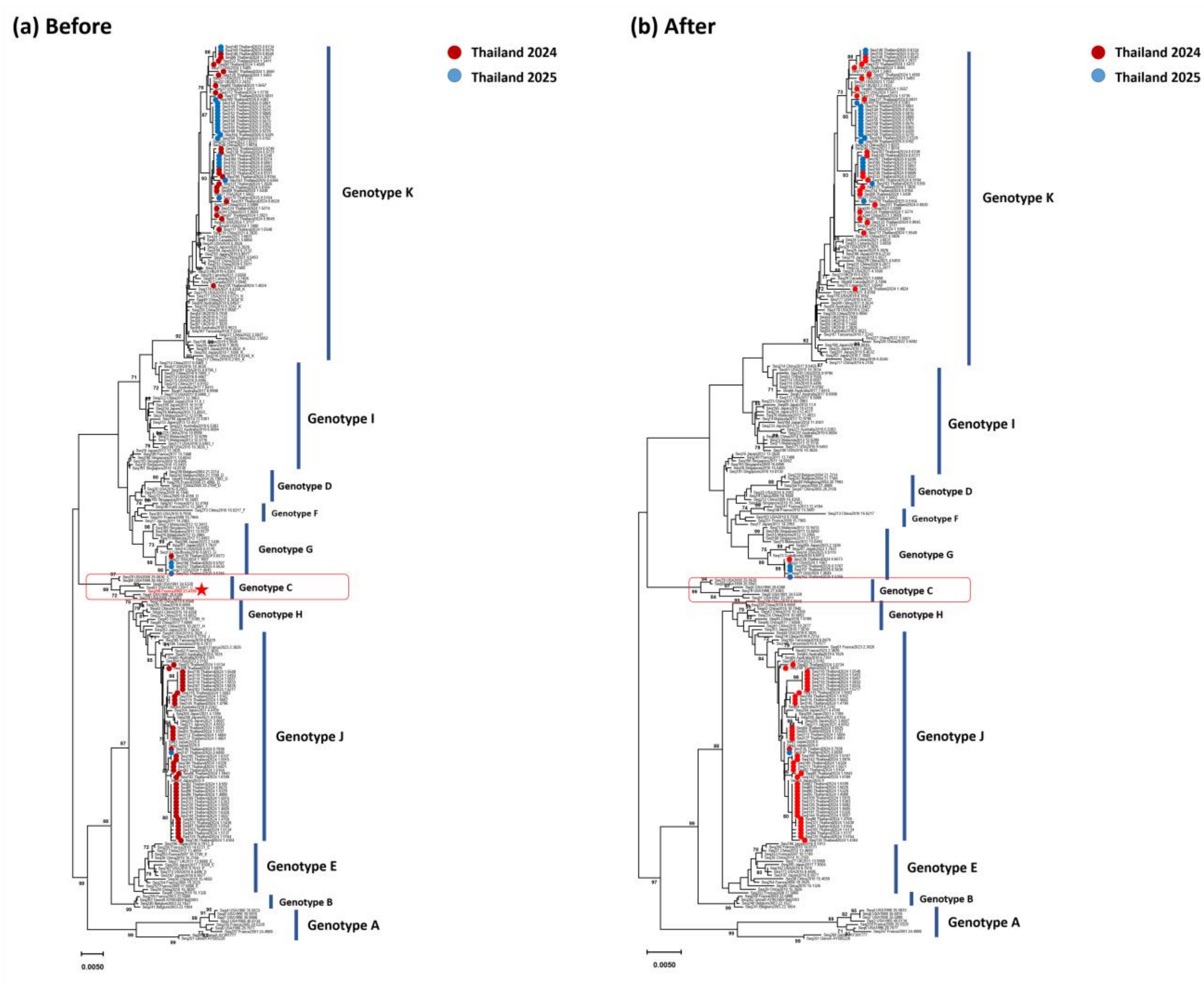

Supplementary Figure 6. Temporal signal assessed by root-to-tip regression. Root-to-tip genetic distances from the maximum-likelihood tree were regressed against sampling dates using TempEST for (a) HCoV-NL63 and (b) HCoV-OC43 partial spike (S) gene sequences. Points represent individual sequences; the solid line denotes the best-fit linear regression and the shaded band indicates the 95% confidence interval.

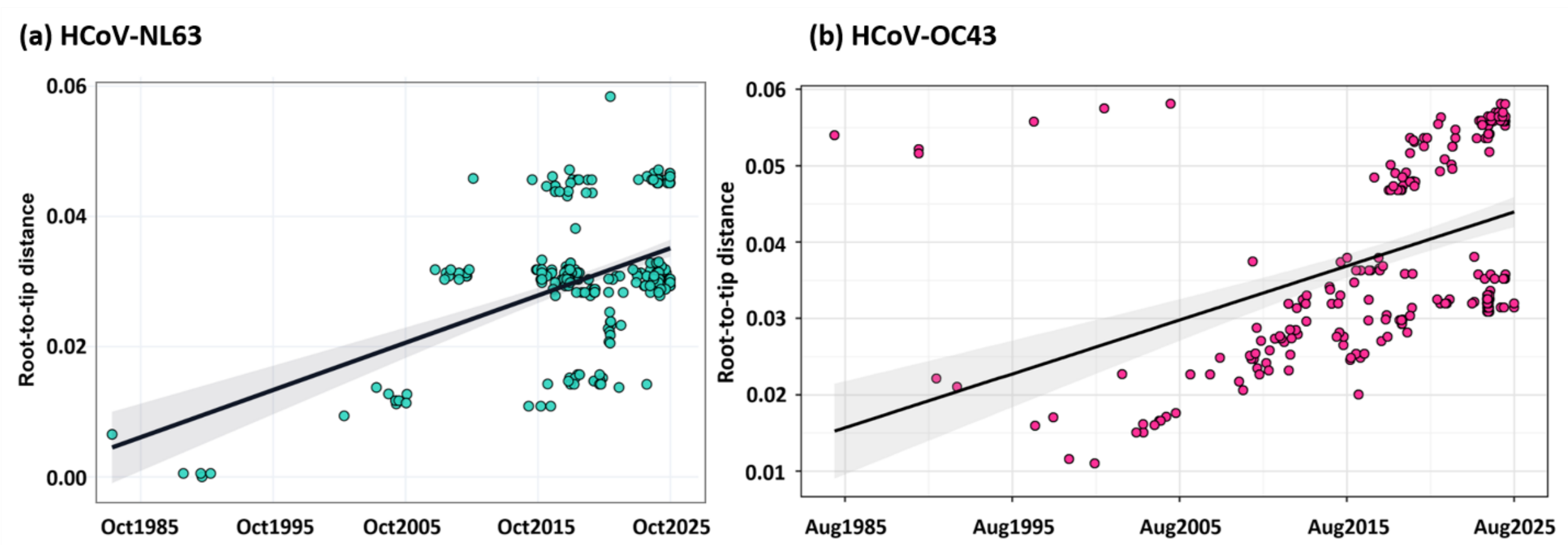

Supplementary Table 3. Markov jump summaries for bidirectional transitions involving Thailand (HCoV-NL63). Values are posterior summaries from Markov jump counts (mean, median, and 95% HPD interval). Net values are calculated as In minus Out, summarized from the posterior distribution.

Region	In mean	In median	In lower	In upper	Out mean	Out median	Out lower	Out upper	Net mean	Net median	Net lower	Net upper
Europe	9.565	11	0	15	0.483	0	0	4	9.082	11	-5	15
China	1.213	0	0	5	0.003	0	0	0	1.210	0	0	5
USA	1.029	0	0	8	8.138	8	2	13	-7.109	-8	-16	3
Japan	0.782	0	0	6	0.073	0	0	0	0.709	0	-2	6
Australia	0.083	0	0	1	0.013	0	0	0	0.070	0	0	1
Asia	0.053	0	0	0	0.280	0	0	1	-0.226	0	-2	0
Russia	0.029	0	0	0	0.009	0	0	0	0.020	0	0	0
Africa	0.009	0	0	0	0.043	0	0	0	-0.034	0	0	0

Supplementary Table 4. Markov jump summaries for bidirectional transitions involving Thailand (HCoV-OC43). Values are posterior summaries from Markov jump counts (mean, median, and 95% HPD interval). Net values are calculated as In minus Out, summarized from the posterior distribution.

Region	In mean	In median	In lower	In upper	Out mean	Out median	Out lower	Out upper	Net mean	Net median	Net lower	Net upper
USA	5.863	6	3	9	4.787	5	2	8	1.076	1	-3	5
Japan	0.450	0	0	2	0.529	0	0	2	-0.078	0	-1	1
China	0.319	0	0	2	0.088	0	0	1	0.231	0	0	2
Europe	0.064	0	0	0	0.215	0	0	2	-0.150	0	-2	0
Canada	0.043	0	0	0	0.071	0	0	0	-0.029	0	-1	0
Australia	0.032	0	0	0	0.009	0	0	0	0.024	0	0	0
Asia	0.002	0	0	0	0.002	0	0	0	0.000	0	0	0
Africa	0.002	0	0	0	0.000	0	0	0	0.001	0	0	0

Supplementary Table 5. Amino-acid substitution enrichment results for HCoV-NL63 (Thailand vs non-Thailand). Results are restricted to substitutions observed ≥3 times across the dataset (total alternate count ≥ 3).

Mutation (AA)	Position (AA)	Ref AA	Alt AA	Thailand: Alt count	Thailand: N (valid)	Non-Thailand: Alt count	Non-Thailand: N (valid)	Total Alt count	Odds ratio (TH vs non-TH)	log2(OR)	P value (Fisher's exact)	FDR q value (BH)	Thailand: Alt frequency (%)	Non-Thailand: Alt frequency (%)
V76I	76	V	I	0	110	49	185	49	0	-6.325	1.14E-11	2.73E-10	0.00	26.49
A280E	280	A	E	0	110	41	185	41	0	-5.988	8.87E-10	1.06E-8	0.00	22.16
K240R	240	K	R	16	110	0	185	16	∞	6.017	6.72E-8	5.37E-7	14.55	0.00
T80I	80	T	I	36	110	18	185	54	4.514	2.149	1.66E-6	9.98E-6	32.73	9.73
E179D	179	E	D	40	110	23	185	63	4.025	1.990	3.32E-6	1.59E-5	36.36	12.43
N175D	175	N	D	12	110	1	185	13	22.531	3.964	4.30E-5	1.72E-4	10.91	0.54
A1V	1	A	V	0	110	21	185	21	0	-4.852	6.49E-5	2.22E-4	0.00	11.35
A12S	12	A	S	35	110	25	185	60	2.987	1.565	2.80E-4	8.41E-4	31.82	13.51
N286S	286	N	S	5	110	0	185	5	∞	4.274	6.80E-3	1.81E-2	4.55	0.00
H211R	211	H	R	0	110	10	185	10	0	-3.725	1.53E-2	3.66E-2	0.00	5.41
V239I	239	V	I	5	110	1	185	6	8.762	2.681	2.85E-2	6.22E-2	4.55	0.54
V129A	129	V	A	0	110	7	185	7	0	-3.215	4.85E-2	8.74E-2	0.00	3.78
V15L	15	V	L	0	110	7	185	7	0	-3.215	4.85E-2	8.74E-2	0.00	3.78
V43L	43	V	L	3	110	0	185	3	∞	3.594	5.10E-2	8.74E-2	2.73	0.00
L215I	215	L	I	39	110	87	185	126	0.619	-0.685	6.77E-2	1.08E-1	35.45	47.03
T479I	479	T	I	0	110	3	185	3	0	-2.084	2.96E-1	4.01E-1	0.00	1.62
I186V	186	I	V	0	110	4	185	4	0	-2.454	3.01E-1	4.01E-1	0.00	2.16
L416V	416	L	V	0	110	4	185	4	0	-2.454	3.01E-1	4.01E-1	0.00	2.16
G242V	242	G	V	1	110	5	185	6	0.330	-1.153	4.17E-1	5.26E-1	0.91	2.70
S429G	429	S	G	36	110	70	185	106	0.799	-0.317	4.52E-1	5.42E-1	32.73	37.84
Q414H	414	Q	H	6	110	7	185	13	1.467	0.566	5.62E-1	6.43E-1	5.45	3.78
L455P	455	L	P	36	110	66	185	102	0.877	-0.184	7.04E-1	7.68E-1	32.73	35.68
H571R	571	H	R	30	110	48	185	78	1.070	0.103	8.91E-1	8.98E-1	27.27	25.95
K139N	139	K	N	37	110	60	185	97	1.056	0.082	8.98E-1	8.98E-1	33.64	32.43

Note: N (valid) is the number of sequences with an unambiguous amino acid at the site. Odds ratios may be 0 or ∞ when one group has zero counts (no continuity correction applied). q values are Benjamini–Hochberg false discovery rate (FDR)–adjusted.

Supplementary Table 6. Amino-acid substitution enrichment results for HCoV-OC43 (Thailand vs non-Thailand). Results are restricted to substitutions observed ≥3 times across the dataset (total alternate count ≥ 3).

Mutation (AA)	Position (AA)	Ref AA	Alt AA	Thailand: Alt count	Thailand: N (valid)	Non-Thailand: Alt count	Non-Thailand: N (valid)	Total Alt count	Odds ratio (TH vs non-TH)	log2(OR)	P value (Fisher's exact)	FDR q value (BH)	Thailand: Alt frequency (%)	Non-Thailand: Alt frequency (%)
N474D	474	N	D	83	89	35	166	118	51.776	5.694	6.61E-31	7.60E-29	93.26	21.08
S16R	16	S	R	1	89	62	159	63	0.018	-5.814	1.66E-13	9.57E-12	1.12	38.99
S495L	495	S	L	0	42	41	67	41	0.008	-7.057	1.61E-12	6.19E-11	0.00	61.19
R423K	423	R	K	32	89	5	166	37	18.077	4.176	3.14E-12	7.65E-11	35.96	3.01
Y564H	564	Y	H	42	89	14	166	56	9.702	3.278	3.99E-12	7.65E-11	47.19	8.43
N23S	23	N	S	42	89	14	166	56	9.702	3.278	3.99E-12	7.65E-11	47.19	8.43
Y186L	186	Y	L	4	89	65	166	69	0.073	-3.774	1.49E-10	2.44E-9	4.49	39.16
D476S	476	D	S	4	89	64	166	68	0.075	-3.737	2.94E-10	3.38E-9	4.49	38.55
A469T	469	A	T	4	89	64	166	68	0.075	-3.737	2.94E-10	3.38E-9	4.49	38.55
K465V	465	K	V	4	89	64	166	68	0.075	-3.737	2.94E-10	3.38E-9	4.49	38.55
R24V	24	R	V	42	89	20	166	62	6.523	2.706	1.49E-9	1.50E-8	47.19	12.05
S16T	16	S	T	42	89	19	159	61	6.585	2.719	1.56E-9	1.50E-8	47.19	11.95
D34E	34	D	E	42	89	21	166	63	6.170	2.625	2.71E-9	2.40E-8	47.19	12.65
R256A	256	R	A	42	89	20	159	62	6.211	2.635	4.78E-9	3.92E-8	47.19	12.58
L57V	57	L	V	42	89	22	166	64	5.849	2.548	7.24E-9	5.55E-8	47.19	13.25
K257D	257	K	D	4	89	55	159	59	0.089	-3.490	9.70E-9	6.97E-8	4.49	34.59
N175K	175	N	K	0	89	39	166	39	0.018	-5.793	1.54E-8	1.04E-7	0.00	23.49
D25Y	25	D	Y	23	89	4	166	27	14.114	3.819	1.83E-8	1.11E-7	25.84	2.41
N142I	142	N	I	20	89	2	166	22	23.768	4.571	1.84E-8	1.11E-7	22.47	1.20
S30N	30	S	N	23	89	4	165	27	14.027	3.810	2.00E-8	1.15E-7	25.84	2.42
R256L	256	R	L	0	89	31	159	31	0.023	-5.455	3.85E-7	2.11E-6	0.00	19.50
P28L	28	P	L	46	89	34	166	80	4.153	2.054	5.32E-7	2.78E-6	51.69	20.48
K257N	257	K	N	42	89	28	159	70	4.181	2.064	1.64E-6	8.21E-6	47.19	17.61
R24I	24	R	I	0	89	29	166	29	0.026	-5.263	1.79E-6	8.49E-6	0.00	17.47
S497G	497	S	G	0	42	24	68	24	0.021	-5.548	1.85E-6	8.49E-6	0.00	35.29
I258H	258	I	H	23	89	7	158	30	7.517	2.910	2.07E-6	9.16E-6	25.84	4.43
R256P	256	R	P	0	89	25	159	25	0.029	-5.085	9.74E-6	4.15E-5	0.00	15.72
D461N	461	D	N	0	89	25	166	25	0.031	-5.012	2.33E-5	9.58E-5	0.00	15.06
K110I	110	K	I	19	88	7	166	26	6.255	2.645	3.61E-5	1.43E-4	21.59	4.22
L154I	154	L	I	19	89	7	166	26	6.165	2.624	3.95E-5	1.51E-4	21.35	4.22
P505S	505	P	S	45	89	39	159	84	3.147	1.654	4.59E-5	1.70E-4	50.56	24.53
P15T	15	P	T	0	47	24	119	24	0.041	-4.607	3.22E-4	1.16E-3	0.00	20.17
F21L	21	F	L	42	89	38	154	80	2.728	1.448	4.00E-4	1.39E-3	47.19	24.68
Y186M	186	Y	M	42	89	42	166	84	2.638	1.400	4.85E-4	1.60E-3	47.19	25.30
D34A	34	D	A	42	89	42	166	84	2.638	1.400	4.85E-4	1.60E-3	47.19	25.30
K18T	18	K	T	42	89	39	149	81	2.520	1.334	1.15E-3	3.68E-3	47.19	26.17
F447L	447	F	L	6	89	0	166	6	25.922	4.696	1.61E-3	4.89E-3	6.74	0.00
K181N	181	K	N	6	89	0	166	6	25.922	4.696	1.61E-3	4.89E-3	6.74	0.00
P28S	28	P	S	0	89	16	166	16	0.051	-4.295	1.69E-3	4.97E-3	0.00	9.64
L17R	17	L	R	42	89	42	156	84	2.426	1.278	1.98E-3	5.69E-3	47.19	26.92

T144I	144	T	I	6	89	0	156	6	24.365	4.607	2.06E-3	5.77E-3	6.74	0.00
I258D	258	I	D	20	89	67	158	87	0.394	-1.345	2.16E-3	5.84E-3	22.47	42.41
L262P	262	L	P	41	89	44	165	85	2.349	1.232	2.18E-3	5.84E-3	46.07	26.67
N525K	525	N	K	5	89	0	166	5	21.675	4.438	4.81E-3	1.23E-2	5.62	0.00
I141V	141	I	V	5	89	0	166	5	21.675	4.438	4.81E-3	1.23E-2	5.62	0.00
T539P	539	T	P	42	89	49	166	91	2.134	1.093	6.11E-3	1.53E-2	47.19	29.52
L529P	529	L	P	46	89	56	166	102	2.101	1.071	7.18E-3	1.76E-2	51.69	33.73
F108I	108	F	I	0	89	11	165	11	0.075	-3.736	9.36E-3	2.23E-2	0.00	6.67
D331N	331	D	N	0	89	11	166	11	0.076	-3.727	9.50E-3	2.23E-2	0.00	6.63
K535T	535	K	T	42	89	47	155	89	2.053	1.038	1.26E-2	2.90E-2	47.19	30.32
D524H	524	D	H	42	89	51	166	93	2.015	1.011	1.38E-2	3.10E-2	47.19	30.72
T528N	528	T	N	42	89	52	166	94	1.959	0.970	1.44E-2	3.19E-2	47.19	31.33
S143L	143	S	L	0	89	10	164	10	0.082	-3.605	1.61E-2	3.50E-2	0.00	6.10
V148D	148	V	D	0	89	10	166	10	0.083	-3.586	1.64E-2	3.50E-2	0.00	6.02
L17Y	17	L	Y	0	89	9	156	9	0.087	-3.527	2.84E-2	5.33E-2	0.00	5.77
R255K	255	R	K	0	89	9	166	9	0.093	-3.433	2.92E-2	5.33E-2	0.00	5.42
F180R	180	F	R	0	89	9	166	9	0.093	-3.433	2.92E-2	5.33E-2	0.00	5.42
D79V	79	D	V	0	89	9	166	9	0.093	-3.433	2.92E-2	5.33E-2	0.00	5.42
T206D	206	T	D	0	89	9	166	9	0.093	-3.433	2.92E-2	5.33E-2	0.00	5.42
T78S	78	T	S	0	89	9	166	9	0.093	-3.433	2.92E-2	5.33E-2	0.00	5.42
I253N	253	I	N	0	89	9	166	9	0.093	-3.433	2.92E-2	5.33E-2	0.00	5.42
L13S	13	L	S	0	89	9	166	9	0.093	-3.433	2.92E-2	5.33E-2	0.00	5.42
N23D	23	N	D	0	89	9	166	9	0.093	-3.433	2.92E-2	5.33E-2	0.00	5.42
S569P	569	S	P	42	89	54	166	96	1.853	0.890	2.97E-2	5.34E-2	47.19	32.53
F228V	228	F	V	42	89	55	166	97	1.803	0.851	3.09E-2	5.47E-2	47.19	33.13
T33I	33	T	I	43	89	57	166	100	1.788	0.838	3.21E-2	5.59E-2	48.31	34.34
S30P	30	S	P	23	89	24	165	47	2.047	1.034	4.12E-2	7.03E-2	25.84	14.55
V463G	463	V	G	3	89	0	166	3	13.474	3.752	4.16E-2	7.03E-2	3.37	0.00
S254R	254	S	R	43	89	58	166	101	1.741	0.800	4.41E-2	7.35E-2	48.31	34.94
V463A	463	V	A	4	89	1	166	5	7.765	2.957	5.17E-2	8.50E-2	4.49	0.60
V157I	157	V	I	0	89	8	166	8	0.104	-3.263	5.35E-2	8.66E-2	0.00	4.82
S420F	420	S	F	42	89	57	166	99	1.709	0.773	5.88E-2	9.40E-2	47.19	34.34
R24K	24	R	K	0	89	6	166	6	0.138	-2.858	9.45E-2	1.47E-1	0.00	3.61
T1M	1	T	M	0	89	7	166	7	0.119	-3.073	9.98E-2	1.47E-1	0.00	4.22
K181T	181	K	T	0	89	7	166	7	0.119	-3.073	9.98E-2	1.47E-1	0.00	4.22
P28V	28	P	V	0	89	7	166	7	0.119	-3.073	9.98E-2	1.47E-1	0.00	4.22
M114L	114	M	L	0	89	7	166	7	0.119	-3.073	9.98E-2	1.47E-1	0.00	4.22
G112D	112	G	D	0	89	7	166	7	0.119	-3.073	9.98E-2	1.47E-1	0.00	4.22
S16K	16	S	K	4	89	17	159	21	0.393	-1.347	1.02E-1	1.49E-1	4.49	10.69
R404G	404	R	G	42	89	60	166	102	1.579	0.659	1.07E-1	1.54E-1	47.19	36.14
E618D	618	E	D	42	89	61	166	103	1.538	0.621	1.10E-1	1.57E-1	47.19	36.75
F597L	597	F	L	42	89	62	166	104	1.499	0.584	1.42E-1	1.92E-1	47.19	37.35
N10K	10	N	K	42	89	62	166	104	1.499	0.584	1.42E-1	1.92E-1	47.19	37.35
N604S	604	N	S	42	89	62	166	104	1.499	0.584	1.42E-1	1.92E-1	47.19	37.35
L80K	80	L	K	42	89	62	166	104	1.499	0.584	1.42E-1	1.92E-1	47.19	37.35

N567G	567	N	G	42	89	62	165	104	1.485	0.570	1.44E-1	1.92E-1	47.19	37.58
Y167H	167	Y	H	4	89	17	166	21	0.412	-1.278	1.52E-1	2.00E-1	4.49	10.24
R256S	256	R	S	1	89	8	159	9	0.214	-2.221	1.63E-1	2.12E-1	1.12	5.03
G506D	506	G	D	0	89	5	165	5	0.163	-2.617	1.66E-1	2.12E-1	0.00	3.03
G566R	566	G	R	0	89	5	166	5	0.164	-2.608	1.66E-1	2.12E-1	0.00	3.01
A486V	486	A	V	4	89	2	166	6	3.859	1.948	1.87E-1	2.37E-1	4.49	1.20
P271S	271	P	S	42	89	66	166	108	1.354	0.437	2.88E-1	3.56E-1	47.19	39.76
K494S	494	K	S	0	42	3	70	3	0.227	-2.140	2.91E-1	3.56E-1	0.00	4.29
T261S	261	T	S	42	89	66	164	108	1.327	0.408	2.91E-1	3.56E-1	47.19	40.24
S30F	30	S	F	0	89	4	165	4	0.200	-2.318	3.01E-1	3.57E-1	0.00	2.42
L586V	586	L	V	0	89	4	166	4	0.202	-2.309	3.01E-1	3.57E-1	0.00	2.41
T539S	539	T	S	0	89	4	166	4	0.202	-2.309	3.01E-1	3.57E-1	0.00	2.41
T83K	83	T	K	4	89	14	166	18	0.511	-0.969	3.10E-1	3.64E-1	4.49	8.43
I322V	322	I	V	9	89	10	166	19	1.755	0.811	3.16E-1	3.68E-1	10.11	6.02
A536G	536	A	G	0	47	4	112	4	0.254	-1.978	3.20E-1	3.68E-1	0.00	3.57
D538G	538	D	G	42	89	68	166	110	1.288	0.365	3.56E-1	4.05E-1	47.19	40.96
F534L	534	F	L	47	89	76	163	123	1.281	0.357	3.59E-1	4.05E-1	52.81	46.63
F472L	472	F	L	42	89	70	166	112	1.226	0.293	5.08E-1	5.68E-1	47.19	42.17
P271L	271	P	L	0	89	3	166	3	0.261	-1.938	5.54E-1	6.01E-1	0.00	1.81
N60K	60	N	K	0	89	3	166	3	0.261	-1.938	5.54E-1	6.01E-1	0.00	1.81
H169Q	169	H	Q	0	89	3	166	3	0.261	-1.938	5.54E-1	6.01E-1	0.00	1.81
I171S	171	I	S	4	89	11	166	15	0.663	-0.593	5.86E-1	6.30E-1	4.49	6.63
P505F	505	P	F	2	89	2	159	4	1.805	0.852	6.20E-1	6.60E-1	2.25	1.26
R71L	71	R	L	1	89	4	166	5	0.460	-1.120	6.61E-1	6.97E-1	1.12	2.41
F108V	108	F	V	4	89	5	165	9	1.506	0.591	7.24E-1	7.57E-1	4.49	3.03
S520Y	520	S	Y	42	89	76	166	118	1.058	0.082	8.95E-1	9.19E-1	47.19	45.78
S545T	545	S	T	42	89	76	166	118	1.058	0.082	8.95E-1	9.19E-1	47.19	45.78
P12T	12	P	T	43	89	79	166	122	1.029	0.042	1.00E+0	1.00E+0	48.31	47.59
T261N	261	T	N	1	89	3	164	4	0.610	-0.713	1.00E+0	1.00E+0	1.12	1.83
G112R	112	G	R	1	89	2	166	3	0.932	-0.102	1.00E+0	1.00E+0	1.12	1.20

Note: N (valid) is the number of sequences with an unambiguous amino acid at the site. Odds ratios may be 0 or ∞ when one group has zero counts (no continuity correction applied). q values are Benjamini–Hochberg false discovery rate (FDR)–adjusted.

Supplementary Table 7. Genotype-stratified enrichment at key HCoV-OC43 spike sites (Thailand vs non-Thailand).

Position	Reference	Alternate	Mutation	Genotype	TH_alt_count	TH_valid_n	NonTH_alt_count	NonTH_valid_n	TH (%)	NonTH (%)	odds_ratio_fisher	p_value	log2OR
16	S	R	S16R	K	1	43	49	77	2.32	63.64	0.014	3.39E-12	-5.620895041
16	S	R	S16R	J	0	42	0	42	0	0	NA	NA	NA
16	S	R	S16R	G	0	4	4	17	0	23.53	0	0.5455305	-1.584962501
21	F	L	F21L	K	0	43	2	79	0	2.53	0	0.5397643	-1.488747185
21	F	L	F21L	J	42	42	36	40	100	90	∞	0.0522509	3.389491379
21	F	L	F21L	G	0	4	0	17	0	0	NA	NA	NA
24	R	V	R24V	K	0	43	0	82	0	0	NA	NA	NA
24	R	V	R24V	J	42	42	13	42	100	30.95	∞	1.73E-12	7.537146483
24	R	V	R24V	G	0	4	0	17	0	0	NA	NA	NA
28	P	L	P28L	K	42	43	28	82	97.67	34.15	81	3.68E-13	5.759722746
28	P	L	P28L	J	0	42	0	42	0	0	NA	NA	NA
28	P	L	P28L	G	4	4	6	17	100	35.29	∞	0.0350877	3.993047239
30	S	N	S30N	K	23	43	4	82	53.49	4.88	22.425	9.56E-10	4.321732595
30	S	N	S30N	J	0	42	0	41	0	0	NA	NA	NA
30	S	N	S30N	G	0	4	0	17	0	0	NA	NA	NA
34	D	E	D34E	K	42	43	21	82	97.67	25.61	122	3.52E-16	6.340678186
34	D	E	D34E	J	0	42	0	42	0	0	NA	NA	NA
34	D	E	D34E	G	0	4	0	17	0	0	NA	NA	NA
143	S	L	S143L	K	0	43	1	82	0	1.22	0	1	-0.679177842
143	S	L	S143L	J	0	42	0	41	0	0	NA	NA	NA
143	S	L	S143L	G	0	4	0	17	0	0	NA	NA	NA

Note: N (valid) is the number of sequences with an unambiguous amino acid at the site within each genotype stratum. Odds ratios (TH vs non-TH) may be 0 or ∞ when one group has zero counts (no continuity correction applied); cells are marked NA when both groups had zero alternate allele counts at that position. P values were calculated using Fisher's exact test; no multiple-testing correction (e.g., FDR) was applied, as this table presents a targeted follow-up analysis of six pre-specified sites identified in S6 Table.
