# Supplementary material for: Spatiotemporal dynamics and phylogeography of HCoV-NL63 and HCoV-OC43 in Thailand, 2024–2025
Source: PLoS One. 2026 Sep 8;21(9):e0357483. doi: 10.1371/journal.pone.0357483 (PMC13552785; doi:10.1371/journal.pone.0357483)
Supplement: Supplementary file 2 [file pone.0357483.s003.docx]

Journal Requirements:

1. When submitting your revision, we need you to address these additional requirements.

Please ensure that your manuscript meets PLOS ONE's style requirements, including those for file naming. The PLOS ONE style templates can be found at https://journals.plos.org/plosone/s/file?id=wjVg/PLOSOne_formatting_sample_main_body.pdf and https://journals.plos.org/plosone/s/file?id=ba62/PLOSOne_formatting_sample_title_authors_affiliations.pdf

Response: We have revised the manuscript to comply with PLOS ONE's formatting requirements, including the style templates for the main body and title/authors/affiliations, as well as file naming conventions.

2. Please note that your Data Availability Statement is currently missing the direct link to access each database. If your manuscript is accepted for publication, you will be asked to provide these details on a very short timeline. We therefore suggest that you provide this information now, though we will not hold up the peer review process if you are unable.

Response: We thank the Editor for pointing this out. We have revised the Data Availability Statement (Lines 232–235) to include the direct link to the database, as follows:

"The sequences generated in this study have been deposited in the NCBI GenBank database (https://www.ncbi.nlm.nih.gov/genbank/) under accession numbers PX987302–PX987411 (HCoV-NL63) and PX987412–PX987500 (HCoV-OC43). A complete list of accession numbers for all sequenced samples is provided in S2 Table."

3. We note that Figures 3c and 4c in your submission contain map images which may be copyrighted. All PLOS content is published under the Creative Commons Attribution License (CC BY 4.0), which means that the manuscript, images, and Supporting Information files will be freely available online, and any third party is permitted to access, download, copy, distribute, and use these materials in any way, even commercially, with proper attribution. For these reasons, we cannot publish previously copyrighted maps or satellite images created using proprietary data, such as Google software (Google Maps, Street View, and Earth). For more information, see our copyright guidelines: http://journals.plos.org/plosone/s/licenses-and-copyright.

We require you to either present written permission from the copyright holder to publish these figures specifically under the CC BY 4.0 license, or remove the figures from your submission:

a. You may seek permission from the original copyright holder of Figures 3c and 4c to publish the content specifically under the CC BY 4.0 license.

We recommend that you contact the original copyright holder with the Content Permission Form (http://journals.plos.org/plosone/s/file?id=7c09/content-permission-form.pdf) and the following text:

“I request permission for the open-access journal PLOS ONE to publish XXX under the Creative Commons Attribution License (CCAL) CC BY 4.0 (http://creativecommons.org/licenses/by/4.0/). Please be aware that this license allows unrestricted use and distribution, even commercially, by third parties. Please reply and provide explicit written permission to publish XXX under a CC BY license and complete the attached form.”

Please upload the completed Content Permission Form or other proof of granted permissions as an "Other" file with your submission.

In the figure caption of the copyrighted figure, please include the following text: “Reprinted from [ref] under a CC BY license, with permission from [name of publisher], original copyright [original copyright year].”

b. If you are unable to obtain permission from the original copyright holder to publish these figures under the CC BY 4.0 license or if the copyright holder’s requirements are incompatible with the CC BY 4.0 license, please either i) remove the figure or ii) supply a replacement figure that complies with the CC BY 4.0 license. Please check copyright information on all replacement figures and update the figure caption with source information. If applicable, please specify in the figure caption text when a figure is similar but not identical to the original image and is therefore for illustrative purposes only.

The following resources for replacing copyrighted map figures may be helpful:

USGS National Map Viewer (public domain): http://viewer.nationalmap.gov/viewer/

The Gateway to Astronaut Photography of Earth (public domain): http://eol.jsc.nasa.gov/sseop/clickmap/

Maps at the CIA (public domain): https://www.cia.gov/library/publications/the-world-factbook/index.html and https://www.cia.gov/library/publications/cia-maps-publications/index.html

NASA Earth Observatory (public domain): http://earthobservatory.nasa.gov/

Landsat: http://landsat.visibleearth.nasa.gov/

USGS EROS (Earth Resources Observatory and Science (EROS) Center) (public domain): http://eros.usgs.gov/#

Natural Earth (public domain): <http://www.naturalearthdata.com/>

Response: We thank the Editor for raising this point. We would like to clarify that Figures 3c and 4c were not created using Google Maps, Google Earth, or any other proprietary mapping service. Both maps were generated de novo in R using the rnaturalearth package (version 1.2.0), which sources country boundary data from Natural Earth (https://www.naturalearthdata.com), a public domain map dataset that explicitly states no permission is required for use, reproduction, or redistribution, including for commercial purposes.

We note that PLOS journals have previously published phylogeographic migration maps generated using this same approach; for example, Lu et al. (PLOS Pathogens, 2024, https://doi.org/10.1371/journal.ppat.1011880) used the R package "rnaturalearth" to construct similar country-level transmission maps, with the following attribution provided in the figure legend: "The European shapefile was created using the R package 'rnaturalearth' (https://cran.r-project.org/web/packages/rnaturalearth/)."

Following this precedent, we have added an equivalent attribution statement to the legends of Figures 3c and 4c in the revised manuscript, as follows:

"The base map was generated using the R package 'rnaturalearth' (version 1.2.0; https://cran.r-project.org/package=rnaturalearth), which sources publicly available Natural Earth data."

We have also updated the Materials and methods section (Bayesian phylogeography subsection) accordingly, which now reads: "...processed in R software (version 4.4.2) [30], which was also used to generate all phylogeographic visualizations, including base maps constructed with the 'rnaturalearth' package (version 1.2.0) sourcing Natural Earth data..."

As no copyrighted or proprietary map content was used, no further permission or Content Permission Form is required for these figures.

4. If the reviewer comments include a recommendation to cite specific previously published works, please review and evaluate these publications to determine whether they are relevant and should be cited. There is no requirement to cite these works unless the editor has indicated otherwise.

Response: We thank the Editor for this note. We have carefully reviewed Reviewer #1's comments and confirm that no specific previously published works were recommended for citation. Therefore, no additional references have been added in response to this requirement.

[Note: HTML markup is below. Please do not edit.]

Reviewers' comments:

Reviewer's Responses to Questions

Comments to the Author

1. Is the manuscript technically sound, and do the data support the conclusions?

The manuscript must describe a technically sound piece of scientific research with data that supports the conclusions. Experiments must have been conducted rigorously, with appropriate controls, replication, and sample sizes. The conclusions must be drawn appropriately based on the data presented.

Reviewer #1: Yes

2. Has the statistical analysis been performed appropriately and rigorously?

Reviewer #1: Yes

3. Have the authors made all data underlying the findings in their manuscript fully available?

The PLOS Data policy requires authors to make all data underlying the findings described in their manuscript fully available without restriction, with rare exception (please refer to the Data Availability Statement in the manuscript PDF file). The data should be provided as part of the manuscript or its supporting information, or deposited to a public repository. For example, in addition to summary statistics, the data points behind means, medians and variance measures should be available. If there are restrictions on publicly sharing data—e.g. participant privacy or use of data from a third party—those must be specified.

Reviewer #1: No

Response: We have expanded the Data Availability Statement to include the underlying raw data supporting the phylogenetic and phylogeographic findings reported in this manuscript. In addition to the GenBank accession numbers already provided, we have deposited the BEAST XML input files, MCMC log files, posterior tree files, MCC/summary tree files, Markov jump results from the discrete-trait phylogeographic analyses, and the R scripts used for data processing and map visualization to a public repository (Figshare).

The revised Data Availability Statement (Lines 232–235) now reads as follows:

"The sequences generated in this study have been deposited in the NCBI GenBank database (https://www.ncbi.nlm.nih.gov/genbank/) under accession numbers PX987302–PX987411 (HCoV-NL63) and PX987412–PX987500 (HCoV-OC43). A complete list of accession numbers for all sequenced samples is provided in S2 Table. Other data, including BEAST XML input files, MCMC log files, posterior tree files, MCC/summary tree files, Markov jump results from discrete-trait phylogeographic analyses, and R scripts used for data processing and map visualization, are available via Figshare at https://doi.org/10.6084/m9.figshare.32967941.

4. Is the manuscript presented in an intelligible fashion and written in standard English?

PLOS ONE does not copyedit accepted manuscripts, so the language in submitted articles must be clear, correct, and unambiguous. Any typographical or grammatical errors should be corrected at revision, so please note any specific errors here.

Reviewer #1: Yes

5. Review Comments to the Author

Please use the space provided to explain your answers to the questions above. You may also include additional comments for the author, including concerns about dual publication, research ethics, or publication ethics. (Please upload your review as an attachment if it exceeds 20,000 characters)

Reviewer #1: The manuscript entitled “Spatiotemporal dynamics and phylogeography of HCoV-NL63 and HCoV-OC43 in Thailand, 2024–2025” presents an integrated molecular epidemiological and phylogeographic analysis of endemic human coronaviruses circulating in Thailand during the post-COVID-19 era. The study combines routine acute respiratory infection (ARI) surveillance with partial spike gene sequencing, Bayesian phylogenetic reconstruction, phylogeographic inference, and codon-based selection analyses to characterize the transmission dynamics and evolutionary patterns of HCoV-NL63 and HCoV-OC43. The findings provide useful information on seasonality, genotype distribution, viral migration patterns, and evolutionary dynamics. However, several methodological, interpretative, and presentation-related issues should be addressed before the manuscript can be considered for publication.

5.1. The manuscript title in the main text is inconsistent with the title provided in the supplementary files. Please ensure that the titles are fully consistent across all submitted materials.

Response:

We apologize for this oversight. We have corrected the title in the supplementary files to match the title of the main manuscript exactly. The title now reads consistently as "Spatiotemporal dynamics and phylogeography of HCoV-NL63 and HCoV-OC43 in Thailand, 2024–2025" across all submitted materials, including the main text and all supplementary files.

5.2. The manuscript would benefit from a more detailed description of the surveillance results across 2024–2025. Specifically, please provide the numbers and proportions of positive samples for all four endemic human coronaviruses (HCoV-OC43, HCoV-NL63, HCoV-HKU1, and HCoV-229E) collected during the study period in Thailand.

Response:

We have revised the Results section to provide the numbers and proportions of positive samples for all four endemic HCoVs (HCoV-OC43, HCoV-NL63, HCoV-HKU1, and HCoV-229E) for both the overall study period and each individual year. We have also added a new supplementary figure (S2 Fig) illustrating the monthly positivity trends for HCoV-HKU1 and HCoV-229E, which were not previously shown, to complement the existing genotype composition data for HCoV-NL63 and HCoV-OC43 (Fig 1b–1c). All subsequent Supplementary figures have been renumbered accordingly.

The revised Results section now reads:

- Lines 245-249;

*"Across the two years combined, 460 HCoV-positive samples were identified from 11,709 ARI hospitalizations screened (3.93%). By species, HCoV-OC43 was the most frequently detected (n = 163, 35.4% of positives), followed by HCoV-NL63 (n = 154, 33.5%), HCoV-HKU1 (n = 77, 16.7%), and HCoV-229E (n = 66, 14.3%)."*

- Lines 264-275;

*"Monthly genotype composition (Fig 1b–1c) suggested seasonal shifts in dominant lineages. In 2024, HCoV-OC43 accounted for the largest annual share (n = 115, 35.0% of 329 positives that year) and contributed a substantial proportion of detections early in the year, whereas HCoV-NL63 (n = 97, 29.5%) increased and predominated during the middle-to-late months; HCoV-HKU1 (n = 70, 21.3%) and HCoV-229E (n = 47, 14.3%) were detected at lower, fluctuating levels throughout the year (S2 Fig). In 2025, total HCoV detections declined (n = 131); HCoV-NL63 became the most frequently detected species overall (n = 55, 42.0%), increasing in relative contribution during the second half of the year and aligning with the late-season spikes observed in the weekly series, while HCoV-OC43 remained substantial (n = 51, 38.9%) and dominant in the early months, consistent with the early-year wave. HCoV-229E (n = 19, 14.5%) and HCoV-HKU1 (n = 6, 4.6%) contributed smaller proportions in 2025, with monthly trends shown in S2 Fig. "*

In addition, please include a more detailed description of the surveillance design, including the sampling strategy, study population, case definitions, and other relevant information.

Response:

We have expanded the Materials and methods section (Lines 117–125) to provide a more detailed description of the surveillance design, including the case definition of ARI, the study population, and the sample collection procedure.

The revised text now reads as follows (Lines 117–125):

*"During 2024–2025, we analyzed 11,709 hospitalized acute respiratory infection (ARI) specimens collected at a collaborating tertiary-care hospital in Bangkok, Thailand, as part of an ongoing respiratory virus surveillance program. ARI was defined as a body temperature ≥38°C accompanied by cough, with symptom onset within the preceding 10 days. Patients of all ages presenting with ARI who were hospitalized were eligible for enrollment. Nasopharyngeal swabs were collected in universal transport medium and transported to our center and processed using a routine diagnostic workflow that screens a broad panel of respiratory pathogens by virus-specific real-time RT-PCR assays (e.g., influenza A/B, RSV, SARS-CoV-2, parainfluenza viruses [PIV1–4], adenovirus, and rhinovirus)."*

5.3. The manuscript states that a subset of coronavirus-positive specimens was randomly selected for partial spike sequencing (Lines 145–146). However, no further details are provided regarding this “random selection”. The authors should clarify how randomization was implemented, whether sequencing selection was stratified by month, Ct value, hospital, age, or viral species, and whether any unsuccessful sequencing attempts occurred. This information is important, as these factors could introduce bias into genotype composition and seasonal lineage interpretation.

Response:

We appreciate the reviewer's comment for raising this important point. We wish to clarify that specimen selection for sequencing was not a random subsample, but rather an exhaustive selection based on a pre-defined Ct value threshold. Specifically, among the 154 HCoV-NL63–positive and 163 HCoV-OC43–positive specimens identified by real-time RT-PCR, all specimens with Ct < 30 (indicating sufficient viral load for reliable sequencing) were selected for partial spike gene sequencing. This yielded 110 HCoV-NL63 specimens and 89 HCoV-OC43 specimens, all of which were successfully sequenced with no sequencing failures. Specimens with Ct ≥ 30 were excluded due to insufficient viral load for reliable amplification. We have corrected the terminology in the manuscript (originally described as "randomly selected") to accurately reflect this Ct-based selection procedure, and have clarified that selection was not further stratified by collection month, hospital, or patient age.

The revised section now reads (Lines 142-148):

*"For partial spike gene sequencing, both HCoV-NL63 and HCoV-OC43-positive specimens with a real-time RT-PCR Ct value < 30 (indicating sufficient viral load for reliable sequencing) were selected for sequencing. Of 154 HCoV-NL63–positive and 163 HCoV-OC43–positive specimens identified by real-time RT-PCR, 110 and 89 specimens, respectively, met the Ct < 30 threshold and were selected for sequencing; all were successfully sequenced, with no sequencing failures. Specimens with Ct ≥ 30 were excluded due to insufficient viral load for reliable amplification and were not eligible for sequencing (S1 Fig)."*

5.4. This study exclusively relies on partial spike gene sequences for genotype assignment, phylogeographic inference, recombination screening, and selection pressure analyses. The manuscript may at times overinterpret findings derived from partial sequences. For example, phylogeographic connectivity, lineage persistence, recombination dynamics, and adaptive evolution are discussed in a manner that may imply genome-wide inference. Because HCoV-OC43 evolution is known to be strongly influenced by recombination, partial S-gene data may not accurately represent genome-wide phylogenetic relationships. The authors should more explicitly state the nucleotide sequence length and genomic coordinates, whether the sequenced region overlaps highly variable or recombination-prone domains, and how partial-sequence limitations may bias genotype assignment and migration inference. The authors should also clarify whether genotype definitions used in this manuscript are strictly spike-based or inferred relative to whole-genome-defined reference genotypes.

We thank the Reviewer for this detailed and important comment. We have addressed each point as follows:

Comment: "*This study exclusively relies on partial spike gene sequences for genotype assignment, phylogeographic inference, recombination screening, and selection pressure analyses. The manuscript may at times overinterpret findings derived from partial sequences. For example, phylogeographic connectivity, lineage persistence, recombination dynamics, and adaptive evolution are discussed in a manner that may imply genome-wide inference.*"

Response: We thank the Reviewer for this important observation. We have revised the Results and Discussion sections (Lines 317–319, 359–363, 379–383, 406–408, 485, 488–491, 558–564) to explicitly qualify statements regarding phylogeographic connectivity, lineage persistence, recombination screening, and adaptive evolution as being based on the partial spike region analyzed, rather than implying genome-wide inference. For example:

- Line 317–319 now reads: *"As this screening was restricted to the sequenced partial spike region, recombination breakpoints occurring elsewhere in the genome cannot be excluded."*
- Line 359–363 now reads: *"Based on this partial-spike discrete-trait analysis, Thailand showed apparent bidirectional connectivity among supported links, with the most prominent inbound route from Europe (Europe–Thailand: BF = 62; p = 0.89) and the strongest outbound route to the USA (Thailand–USA: BF = 157; p = 0.95)."*
- Line 379–383 now reads: *"The scattering of Thai tips among global references (n = 163), together with the presence of multiple Thai subclusters, is most consistent with repeated introductions into Thailand followed by local onward transmission in more than one lineage, based on the partial spike topology; because HCoV-OC43 evolution is influenced by recombination, this inference may not fully reflect genome-wide transmission history."*
- Line 406–408 now reads: *"The inferred diffusion network, based on the sequenced partial spike region, showed broad transcontinental connectivity, with prominent links among the USA, China, and Europe."*

Comment: "*The authors should more explicitly state the nucleotide sequence length and genomic coordinates, whether the sequenced region overlaps highly variable or recombination-prone domains, and how partial-sequence limitations may bias genotype assignment and migration inference*."

Response: We have added the nucleotide sequence length and spike protein domain coverage for both species in the Materials and methods section (Lines 151-157): HCoV-NL63 (1,875 nt; residues <1–624, covering the S1 C-terminus, S1/S2 cleavage region, and S2 fusion machinery) and HCoV-OC43 (1,842 nt; residues 6–611, covering the N-terminal domain and receptor-binding domain).

The revised text now reads as follows (Lines 151-157):

"*This approach yielded amplicons of 1,875 nucleotides for HCoV-NL63 and 1,842 nucleotides for HCoV-OC43. Based on alignment to reference spike protein domains, the HCoV-NL63 amplicon (encoding residues <1–624) spans the C-terminal portion of the S1 subunit, the S1/S2 cleavage region, and the N-terminal portion of the S2 subunit, including the fusion peptide and internal fusion peptide. The HCoV-OC43 amplicon (encoding residues 6–611) spans the N-terminal domain and the majority of the receptor-binding domain (RBD) of the S1 subunit, including the receptor-binding motif*."

We have also clarified in the Limitations sections that the HCoV-OC43 sequenced region predominantly covers the receptor-binding domain (RBD), a region subject to strong immune-driven diversifying selection and implicated as recombination-prone in other coronaviruses, whereas the HCoV-NL63 region covers the more conserved S1/S2 and S2 fusion machinery.

The revised text now reads as follows (Lines 571-589):

*"The sequenced regions differed in their coverage of spike protein domains between the two species: the HCoV-NL63 partial sequence (1,875 nucleotides; 624 amino acids) covers the C-terminal portion of the S1 subunit, the S1/S2 cleavage region, and the N-terminal portion of the S2 subunit (including the fusion peptide), but does not include the receptor-binding domain (RBD); in contrast, the HCoV-OC43 partial sequence (1,842 nucleotides; 614 amino acids) encompasses the N-terminal domain and nearly the entirety of the RBD, including the receptor-binding motif, but does not extend into the S2 subunit. Because the RBD is subject to strong immune-driven diversifying selection and has been implicated as a recombination-prone region in other coronaviruses, genotype assignments and selection pressure analyses for HCoV-OC43 may be disproportionately influenced by evolutionary processes specific to this domain, whereas the HCoV-NL63 analysis, covering the more conserved S1/S2 and S2 fusion machinery, may better reflect genome-wide relationships but underrepresent RBD-driven diversification. Because tree topology and inferred migration routes are estimated from the sequenced locus, the same domain-specific constraints could also influence phylogeographic and migration inferences if recombination has decoupled the evolutionary history of the RBD from that of the rest of the genome, particularly for HCoV-OC43. These domain-specific differences should be considered when interpreting genotype assignments and phylogeographic inferences for each species, and genome-wide sequencing would be needed to fully resolve these limitations."*

Comment: *"The authors should also clarify whether genotype definitions used in this manuscript are strictly spike-based or inferred relative to whole-genome-defined reference genotypes."*

Response: We have clarified in the Results (Lines 292-297) that genotype assignments for both HCoV-NL63 and HCoV-OC43 were made relative to previously established, whole-genome-informed reference genotype classifications [7,16-17], rather than being newly defined from our partial spike data. Thai sequences were placed into these established genotype clades based on their phylogenetic position within maximum-likelihood trees constructed using our partial spike alignment together with global reference sequences.

The revised text now reads as follows (Lines 292-297):

*"Genotype assignments for HCoV-NL63 and HCoV-OC43 in this study were made relative to previously established, whole-genome-informed reference genotype classifications (HCoV-OC43, genotypes A–K [7]; HCoV-NL63, genotypes A–C [16-17]), rather than being newly defined from the partial spike sequences generated in this study. Thai sequences were placed into these reference genotype clades based on their phylogenetic position within maximum-likelihood trees constructed using the partial spike alignment together with global reference sequences representing each established genotype (S3 Fig)."*

5.5. In the figure legend of Figure 1 and in the corresponding Results section (Lines 255–266), the term “genotype” is used in a misleading way to describe both HCoV-OC43 and HCoV-NL63. This usage is not appropriate. The term “genotype” should only refer to genetic variants within a single coronavirus species (e.g., different genotypes of HCoV-OC43 such as A–K), rather than being used to compare or describe different human coronavirus species.

Response: We thank the Reviewer for pointing out this terminological inconsistency. We agree that "genotype" should not be used to compare or describe different HCoV species, and have corrected the terminology in both the Figure 1 legend and the corresponding Results section.

The Figure 1 legend has been revised (now reads):

*"Fig 1. Weekly coronavirus positivity and species/genotype in Thailand, 2024–2025. (a) Weekly percent positivity shown as 5-week weighted moving average (WMA5) for endemic HCoVs (solid line) and SARS-CoV-2 (dashed line); bars indicate total ARI specimens tested (right axis). (b–c) Monthly genotype composition within HCoV-NL63 and HCoV-OC43, respectively."*

Moreover, in the Results section (Line 279), *"Monthly genotype composition..."* has been changed to *"Monthly species composition..."* to correctly reflect that this sentence compares detection patterns across different HCoV species (HCoV-OC43, HCoV-NL63, HCoV-HKU1, and HCoV-229E), rather than genotypes within a single species. The term "genotype" has been retained only where it correctly refers to genetic variants within a single species (e.g., genotype J, K within HCoV-OC43; genotype C4, B2, C3 within HCoV-NL63).

5.6. In the Supplementary Table 7 and in the corresponding Results section (Lines 435–441), the analysis describes amino acid mutations in the spike protein of HCoV-OC43. However, both the main text and Table S7 appear to annotate HCoV-OC43 variants using genotype labels such as C2 and C3, which are typically associated with HCoV-NL63. In contrast, HCoV-OC43 genotypes are currently classified as A–K. This inconsistency in genotype annotation should be carefully corrected. And, the footnotes of the tables should also be carefully reviewed to ensure accuracy and consistency.

Response: We thank the Reviewer for catching this important inconsistency. We confirm that this was an annotation error: the genotype labels in Supplementary Table 7 and the corresponding Results text had been mistakenly carried over from the HCoV-NL63 genotype nomenclature (A–C system) instead of the correct HCoV-OC43 genotype nomenclature (A–K system). We have corrected all affected genotype labels in Supplementary Table 7 (C3 → K, D → J, C2 → G, based on matching sample sizes with the genotype distribution reported in the Results) and in the corresponding Discussion text (Lines 476–481).

The revised text now reads as follows (Lines 476–481):

*"Within genotype K, positions 16, 28, 30, and 34 remained strongly differentiated between Thai and non-Thai sequences, indicating that these patterns were not explained by genotype composition alone. Position 24 was largely linked to genotype J but still differed within J, whereas position 143 was rare in major genotypes and its apparent enrichment was driven by minor non-Thai genotypes (S7 Table)."*

We have also reviewed the footnotes of Supplementary Table 7, as well as all other supplementary tables (S3–S6), to ensure consistent and accurate genotype terminology throughout.

5.7. In the references section, reference number 29 is incorrectly placed on a separate line, while the corresponding citation appears on the following line (Lines 634–636). Please correct this formatting issue and ensure proper reference formatting consistency.

Response: We thank the Reviewer for catching this formatting error. We have corrected reference 29, which was inadvertently split across two lines due to a line-break error, so that the reference number and its corresponding citation now appear together on the same line, consistent with the formatting of all other references in the list. We have also reviewed the entire reference list to confirm consistent formatting throughout.

6. PLOS authors have the option to publish the peer review history of their article (what does this mean?). If published, this will include your full peer review and any attached files.

If you choose “no”, your identity will remain anonymous but your review may still be made public.

Do you want your identity to be public for this peer review? For information about this choice, including consent withdrawal, please see our Privacy Policy.

Yes: Chenglong Xiong
