# Supplementary material for: Spatiotemporal dynamics and phylogeography of HCoV-NL63 and HCoV-OC43 in Thailand, 2024–2025
Source: PLoS One. 2026 Sep 8;21(9):e0357483. doi: 10.1371/journal.pone.0357483 (PMC13552785; doi:10.1371/journal.pone.0357483)
Supplement: Peer review history

# Peer review: Decision Letter 0

**DOI:** 10.1371/journal.pone.0357483.r001

**Authors:** Haitham Amer

10 Jul 2026

Dear Dr. Poovorawan,

Thank you for submitting your manuscript to PLOS ONE. After careful consideration, we feel that it has merit but does not fully meet PLOS ONE’s publication criteria as it currently stands. Therefore, we invite you to submit a revised version of the manuscript that addresses the points raised during the review process.

Please submit your revised manuscript by Aug 24 2026 11:59PM. If you will need more time than this to complete your revisions, please reply to this message or contact the journal office at plosone@plos.org. When you're ready to submit your revision, log on to https://www.editorialmanager.com/pone/ and select the 'Submissions Needing Revision' folder to locate your manuscript file.

- A letter that responds to each point raised by the academic editor and reviewer(s). You should upload this letter as a separate file labeled 'Response to Reviewers'.
- A marked-up copy of your manuscript that highlights changes made to the original version. You should upload this as a separate file labeled 'Revised Manuscript with Track Changes'.
- An unmarked version of your revised paper without tracked changes. You should upload this as a separate file labeled 'Manuscript'.

If you would like to make changes to your financial disclosure, please include your updated statement in your cover letter. Guidelines for resubmitting your figure files are available below the reviewer comments at the end of this letter.

If applicable, we recommend that you deposit your laboratory protocols in protocols.io to enhance the reproducibility of your results. Protocols.io assigns your protocol its own identifier (DOI) so that it can be cited independently in the future. For instructions see: https://journals.plos.org/plosone/s/submission-guidelines#loc-laboratory-protocols. Additionally, PLOS ONE offers an option for publishing peer-reviewed Lab Protocol articles, which describe protocols hosted on protocols.io. Read more information on sharing protocols at https://plos.org/protocols?utm_medium=editorial-email&utm_source=authorletters&utm_campaign=protocols.

As the corresponding author, your ORCID iD is verified in the submission system and will appear in the published article. PLOS supports the use of ORCID, and we encourage all coauthors to register for an ORCID iD and use it as well. Please encourage your coauthors to verify their ORCID iD within the submission system before final acceptance, as unverified ORCID iDs will not appear in the published article. *Only*  the individual author can complete the verification step; PLOS staff *cannot*  verify ORCID iDs on behalf of authors.

We look forward to receiving your revised manuscript.

Kind regards,

Haitham Mohamed Amer, PhD

Academic Editor

PLOS One

**Journal Requirements:**

1. When submitting your revision, we need you to address these additional requirements.

Please ensure that your manuscript meets PLOS ONE's style requirements, including those for file naming. The PLOS ONE style templates can be found at

https://journals.plos.org/plosone/s/file?id=wjVg/PLOSOne_formatting_sample_main_body.pdf and

https://journals.plos.org/plosone/s/file?id=ba62/PLOSOne_formatting_sample_title_authors_affiliations.pdf

2. Please note that your Data Availability Statement is currently missing the direct link to access each database. If your manuscript is accepted for publication, you will be asked to provide these details on a very short timeline. We therefore suggest that you provide this information now, though we will not hold up the peer review process if you are unable.

3. We note that Figures 3c and 4c in your submission contain map images which may be copyrighted. All PLOS content is published under the Creative Commons Attribution License (CC BY 4.0), which means that the manuscript, images, and Supporting Information files will be freely available online, and any third party is permitted to access, download, copy, distribute, and use these materials in any way, even commercially, with proper attribution. For these reasons, we cannot publish previously copyrighted maps or satellite images created using proprietary data, such as Google software (Google Maps, Street View, and Earth). For more information, see our copyright guidelines: http://journals.plos.org/plosone/s/licenses-and-copyright.

We require you to either present written permission from the copyright holder to publish these figures specifically under the CC BY 4.0 license, or remove the figures from your submission:

a. You may seek permission from the original copyright holder of Figures 3c and 4c to publish the content specifically under the CC BY 4.0 license.

We recommend that you contact the original copyright holder with the Content Permission Form (http://journals.plos.org/plosone/s/file?id=7c09/content-permission-form.pdf) and the following text:

“I request permission for the open-access journal PLOS ONE to publish XXX under the Creative Commons Attribution License (CCAL) CC BY 4.0 (http://creativecommons.org/licenses/by/4.0/). Please be aware that this license allows unrestricted use and distribution, even commercially, by third parties. Please reply and provide explicit written permission to publish XXX under a CC BY license and complete the attached form.”

Please upload the completed Content Permission Form or other proof of granted permissions as an "Other" file with your submission.

In the figure caption of the copyrighted figure, please include the following text: “Reprinted from [ref] under a CC BY license, with permission from [name of publisher], original copyright [original copyright year].”

b. If you are unable to obtain permission from the original copyright holder to publish these figures under the CC BY 4.0 license or if the copyright holder’s requirements are incompatible with the CC BY 4.0 license, please either i) remove the figure or ii) supply a replacement figure that complies with the CC BY 4.0 license. Please check copyright information on all replacement figures and update the figure caption with source information. If applicable, please specify in the figure caption text when a figure is similar but not identical to the original image and is therefore for illustrative purposes only.

The following resources for replacing copyrighted map figures may be helpful:

USGS National Map Viewer (public domain): http://viewer.nationalmap.gov/viewer/

The Gateway to Astronaut Photography of Earth (public domain): http://eol.jsc.nasa.gov/sseop/clickmap/

Maps at the CIA (public domain): https://www.cia.gov/library/publications/the-world-factbook/index.html and https://www.cia.gov/library/publications/cia-maps-publications/index.html

NASA Earth Observatory (public domain): http://earthobservatory.nasa.gov/

Landsat: http://landsat.visibleearth.nasa.gov/

USGS EROS (Earth Resources Observatory and Science (EROS) Center) (public domain): http://eros.usgs.gov/#

Natural Earth (public domain): http://www.naturalearthdata.com/

4. If the reviewer comments include a recommendation to cite specific previously published works, please review and evaluate these publications to determine whether they are relevant and should be cited. There is no requirement to cite these works unless the editor has indicated otherwise. 

[Note: HTML markup is below. Please do not edit.]

Reviewers' comments:

Reviewer's Responses to Questions

**Comments to the Author**

1. Is the manuscript technically sound, and do the data support the conclusions?

Reviewer #1: Yes

**********

2. Has the statistical analysis been performed appropriately and rigorously?

Reviewer #1: Yes

**********

3. Have the authors made all data underlying the findings in their manuscript fully available?

The PLOS Data policy

Reviewer #1: No

**********

4. Is the manuscript presented in an intelligible fashion and written in standard English?

Reviewer #1: Yes

**********

**Reviewer #1:**  The manuscript entitled “Spatiotemporal dynamics and phylogeography of HCoV-NL63 and HCoV-OC43 in Thailand, 2024–2025” presents an integrated molecular epidemiological and phylogeographic analysis of endemic human coronaviruses circulating in Thailand during the post-COVID-19 era. The study combines routine acute respiratory infection (ARI) surveillance with partial spike gene sequencing, Bayesian phylogenetic reconstruction, phylogeographic inference, and codon-based selection analyses to characterize the transmission dynamics and evolutionary patterns of HCoV-NL63 and HCoV-OC43. The findings provide useful information on seasonality, genotype distribution, viral migration patterns, and evolutionary dynamics. However, several methodological, interpretative, and presentation-related issues should be addressed before the manuscript can be considered for publication.

1. The manuscript title in the main text is inconsistent with the title provided in the supplementary files. Please ensure that the titles are fully consistent across all submitted materials.

2. The manuscript would benefit from a more detailed description of the surveillance results across 2024–2025. Specifically, please provide the numbers and proportions of positive samples for all four endemic human coronaviruses (HCoV-OC43, HCoV-NL63, HCoV-HKU1, and HCoV-229E) collected during the study period in Thailand. In addition, please include a more detailed description of the surveillance design, including the sampling strategy, study population, case definitions, and other relevant information.

3. The manuscript states that a subset of coronavirus-positive specimens was randomly selected for partial spike sequencing (Lines 145–146). However, no further details are provided regarding this “random selection”. The authors should clarify how randomization was implemented, whether sequencing selection was stratified by month, Ct value, hospital, age, or viral species, and whether any unsuccessful sequencing attempts occurred. This information is important, as these factors could introduce bias into genotype composition and seasonal lineage interpretation.

4. This study exclusively relies on partial spike gene sequences for genotype assignment, phylogeographic inference, recombination screening, and selection pressure analyses. The manuscript may at times overinterpret findings derived from partial sequences. For example, phylogeographic connectivity, lineage persistence, recombination dynamics, and adaptive evolution are discussed in a manner that may imply genome-wide inference. Because HCoV-OC43 evolution is known to be strongly influenced by recombination, partial S-gene data may not accurately represent genome-wide phylogenetic relationships. The authors should more explicitly state the nucleotide sequence length and genomic coordinates, whether the sequenced region overlaps highly variable or recombination-prone domains, and how partial-sequence limitations may bias genotype assignment and migration inference. The authors should also clarify whether genotype definitions used in this manuscript are strictly spike-based or inferred relative to whole-genome-defined reference genotypes.

5. In the figure legend of Figure 1 and in the corresponding Results section (Lines 255–266), the term “genotype” is used in a misleading way to describe both HCoV-OC43 and HCoV-NL63. This usage is not appropriate. The term “genotype” should only refer to genetic variants within a single coronavirus species (e.g., different genotypes of HCoV-OC43 such as A–K), rather than being used to compare or describe different human coronavirus species.

6. In the Supplementary Table 7 and in the corresponding Results section (Lines 435–441), the analysis describes amino acid mutations in the spike protein of HCoV-OC43. However, both the main text and Table S7 appear to annotate HCoV-OC43 variants using genotype labels such as C2 and C3, which are typically associated with HCoV-NL63. In contrast, HCoV-OC43 genotypes are currently classified as A–K. This inconsistency in genotype annotation should be carefully corrected. And, the footnotes of the tables should also be carefully reviewed to ensure accuracy and consistency.

7. In the references section, reference number 29 is incorrectly placed on a separate line, while the corresponding citation appears on the following line (Lines 634–636). Please correct this formatting issue and ensure proper reference formatting consistency.

**********

6. PLOS authors have the option to publish the peer review history of their article (what does this mean?). If published, this will include your full peer review and any attached files.

If you choose “no”, your identity will remain anonymous but your review may still be made public.

**Do you want your identity to be public for this peer review?**  For information about this choice, including consent withdrawal, please see our Privacy Policy.

**Yes:**  Chenglong Xiong

**********

[NOTE: If reviewer comments were submitted as an attachment file, they will be attached to this email and accessible via the submission site. Please log into your account, locate the manuscript record, and check for the action link "View Attachments". If this link does not appear, there are no attachment files.]

To ensure your figures meet our technical requirements, please review our figure guidelines: https://journals.plos.org/plosone/s/figures

You may also use PLOS’s free figure tool, NAAS, to help you prepare publication quality figures: https://journals.plos.org/plosone/s/figures#loc-tools-for-figure-preparation.

NAAS will assess whether your figures meet our technical requirements by comparing each figure against our figure specifications.

# Author comment: Author response to Decision Letter 1

**DOI:** 10.1371/journal.pone.0357483.r002

19 Jul 2026

Journal Requirements:

1. When submitting your revision, we need you to address these additional requirements.

Please ensure that your manuscript meets PLOS ONE's style requirements, including those for file naming. The PLOS ONE style templates can be found at https://journals.plos.org/plosone/s/file?id=wjVg/PLOSOne_formatting_sample_main_body.pdf and https://journals.plos.org/plosone/s/file?id=ba62/PLOSOne_formatting_sample_title_authors_affiliations.pdf

Response: We have revised the manuscript to comply with PLOS ONE's formatting requirements, including the style templates for the main body and title/authors/affiliations, as well as file naming conventions.

2. Please note that your Data Availability Statement is currently missing the direct link to access each database. If your manuscript is accepted for publication, you will be asked to provide these details on a very short timeline. We therefore suggest that you provide this information now, though we will not hold up the peer review process if you are unable.

Response: We thank the Editor for pointing this out. We have revised the Data Availability Statement (Lines 232–235) to include the direct link to the database, as follows:

"The sequences generated in this study have been deposited in the NCBI GenBank database (https://www.ncbi.nlm.nih.gov/genbank/) under accession numbers PX987302–PX987411 (HCoV-NL63) and PX987412–PX987500 (HCoV-OC43). A complete list of accession numbers for all sequenced samples is provided in S2 Table."

3. We note that Figures 3c and 4c in your submission contain map images which may be copyrighted. All PLOS content is published under the Creative Commons Attribution License (CC BY 4.0), which means that the manuscript, images, and Supporting Information files will be freely available online, and any third party is permitted to access, download, copy, distribute, and use these materials in any way, even commercially, with proper attribution. For these reasons, we cannot publish previously copyrighted maps or satellite images created using proprietary data, such as Google software (Google Maps, Street View, and Earth). For more information, see our copyright guidelines: http://journals.plos.org/plosone/s/licenses-and-copyright.

We require you to either present written permission from the copyright holder to publish these figures specifically under the CC BY 4.0 license, or remove the figures from your submission:

a. You may seek permission from the original copyright holder of Figures 3c and 4c to publish the content specifically under the CC BY 4.0 license.

We recommend that you contact the original copyright holder with the Content Permission Form (http://journals.plos.org/plosone/s/file?id=7c09/content-permission-form.pdf) and the following text:

“I request permission for the open-access journal PLOS ONE to publish XXX under the Creative Commons Attribution License (CCAL) CC BY 4.0 (http://creativecommons.org/licenses/by/4.0/). Please be aware that this license allows unrestricted use and distribution, even commercially, by third parties. Please reply and provide explicit written permission to publish XXX under a CC BY license and complete the attached form.”

Please upload the completed Content Permission Form or other proof of granted permissions as an "Other" file with your submission.

In the figure caption of the copyrighted figure, please include the following text: “Reprinted from [ref] under a CC BY license, with permission from [name of publisher], original copyright [original copyright year].”

b. If you are unable to obtain permission from the original copyright holder to publish these figures under the CC BY 4.0 license or if the copyright holder’s requirements are incompatible with the CC BY 4.0 license, please either i) remove the figure or ii) supply a replacement figure that complies with the CC BY 4.0 license. Please check copyright information on all replacement figures and update the figure caption with source information. If applicable, please specify in the figure caption text when a figure is similar but not identical to the original image and is therefore for illustrative purposes only.

The following resources for replacing copyrighted map figures may be helpful:

USGS National Map Viewer (public domain): http://viewer.nationalmap.gov/viewer/

The Gateway to Astronaut Photography of Earth (public domain): http://eol.jsc.nasa.gov/sseop/clickmap/

Maps at the CIA (public domain): https://www.cia.gov/library/publications/the-world-factbook/index.html and https://www.cia.gov/library/publications/cia-maps-publications/index.html

NASA Earth Observatory (public domain): http://earthobservatory.nasa.gov/

Landsat: http://landsat.visibleearth.nasa.gov/

USGS EROS (Earth Resources Observatory and Science (EROS) Center) (public domain): http://eros.usgs.gov/#

Natural Earth (public domain): http://www.naturalearthdata.com/

Response: We thank the Editor for raising this point. We would like to clarify that Figures 3c and 4c were not created using Google Maps, Google Earth, or any other proprietary mapping service. Both maps were generated de novo in R using the rnaturalearth package (version 1.2.0), which sources country boundary data from Natural Earth (https://www.naturalearthdata.com), a public domain map dataset that explicitly states no permission is required for use, reproduction, or redistribution, including for commercial purposes.

We note that PLOS journals have previously published phylogeographic migration maps generated using this same approach; for example, Lu et al. (PLOS Pathogens, 2024, https://doi.org/10.1371/journal.ppat.1011880) used the R package "rnaturalearth" to construct similar country-level transmission maps, with the following attribution provided in the figure legend: "The European shapefile was created using the R package 'rnaturalearth' (https://cran.r-project.org/web/packages/rnaturalearth/)."

Following this precedent, we have added an equivalent attribution statement to the legends of Figures 3c and 4c in the revised manuscript, as follows:

"The base map was generated using the R package 'rnaturalearth' (version 1.2.0; https://cran.r-project.org/package=rnaturalearth), which sources publicly available Natural Earth data."

We have also updated the Materials and methods section (Bayesian phylogeography subsection) accordingly, which now reads: "...processed in R software (version 4.4.2) [30], which was also used to generate all phylogeographic visualizations, including base maps constructed with the 'rnaturalearth' package (version 1.2.0) sourcing Natural Earth data..."

As no copyrighted or proprietary map content was used, no further permission or Content Permission Form is required for these figures.

4. If the reviewer comments include a recommendation to cite specific previously published works, please review and evaluate these publications to determine whether they are relevant and should be cited. There is no requirement to cite these works unless the editor has indicated otherwise.

Response: We thank the Editor for this note. We have carefully reviewed Reviewer #1's comments and confirm that no specific previously published works were recommended for citation. Therefore, no additional references have been added in response to this requirement.

[Note: HTML markup is below. Please do not edit.]

Reviewers' comments:

Reviewer's Responses to Questions

Comments to the Author

1. Is the manuscript technically sound, and do the data support the conclusions?

The manuscript must describe a technically sound piece of scientific research with data that supports the conclusions. Experiments must have been conducted rigorously, with appropriate controls, replication, and sample sizes. The conclusions must be drawn appropriately based on the data presented.

Reviewer #1: Yes

2. Has the statistical analysis been performed appropriately and rigorously?

Reviewer #1: Yes

3. Have the authors made all data underlying the findings in their manuscript fully available?

The PLOS Data policy requires authors to make all data underlying the findings described in their manuscript fully available without restriction, with rare exception (please refer to the Data Availability Statement in the manuscript PDF file). The data should be provided as part of the manuscript or its supporting information, or deposited to a public repository. For example, in addition to summary statistics, the data points behind means, medians and variance measures should be available. If there are restrictions on publicly sharing data—e.g. participant privacy or use of data from a third party—those must be specified.

Reviewer #1: No

Response: We have expanded the Data Availability Statement to include the underlying raw data supporting the phylogenetic and phylogeographic findings reported in this manuscript. In addition to the GenBank accession numbers already provided, we have deposited the BEAST XML input files, MCMC log files, posterior tree files, MCC/summary tree files, Markov jump results from the discrete-trait phylogeographic analyses, and the R scripts used for data processing and map visualization to a public repository (Figshare).

The revised Data Availability Statement (Lines 232–235) now reads as follows:

"The sequences generated in this study have been deposited in the NCBI GenBank database (https://www.ncbi.nlm.nih.gov/genbank/) under accession numbers PX987302–PX987411 (HCoV-NL63) and PX987412–PX987500 (HCoV-OC43). A complete list of accession numbers for all sequenced samples is provided in S2 Table. Other data, including BEAST XML input files, MCMC log files, posterior tree files, MCC/summary tree files, Markov jump results from discrete-trait phylogeographic analyses, and R scripts used for data processing and map visualization, are available via Figshare at https://doi.org/10.6084/m9.figshare.32967941.

4. Is the manuscript presented in an intelligible fashion and written in standard English?

PLOS ONE does not copyedit accepted manuscripts, so the language in submitted articles must be clear, correct, and unambiguous. Any typographical or grammatical errors should be corrected at revision, so please note any specific errors here.

Reviewer #1: Yes

5. Review Comments to the Author

Please use the space provided to explain your answers to the questions above. You may also include additional comments for the author, including concerns about dual publication, research ethics, or publication ethics. (Please upload your review as an attachment if it exceeds 20,000 characters)

Reviewer #1: The manuscript entitled “Spatiotemporal dynamics and phylogeography of HCoV-NL63 and HCoV-OC43 in Thailand, 2024–2025” presents an integrated molecular epidemiological and phylogeographic analysis of endemic human coronaviruses circulating in Thailand during the post-COVID-19 era. The study combines routine acute respiratory infection (ARI) surveillance with partial spike gene sequencing, Bayesian phylogenetic reconstruction, phylogeographic inference, and codon-based selection analyses to characterize the transmission dynamics and evolutionary patterns of HCoV-NL63 and HCoV-OC43. The findings provide useful information on seasonality, genotype distribution, viral migration patterns, and evolutionary dynamics. However, several methodological, interpretative, and presentation-related issues should be addressed before the manuscript can be considered for publication.

5.1. The manuscript title in the main text is inconsistent with the title provided in the supplementary files. Please ensure that the titles are fully consistent across all submitted materials.

Response:

We apologize for this oversight. We have corrected the title in the supplementary files to match the title of the main manuscript exactly. The title now reads consistently as "Spatiotemporal dynamics and phylogeography of HCoV-NL63 and HCoV-OC43 in Thailand, 2024–2025" across all submitted materials, including the main text and all supplementary files.

5.2. The manuscript would benefit from a more detailed description of the surveillance results across 2024–2025. Specifically, please provide the numbers and proportions of positive samples for all four endemic human coronaviruses (HCoV-OC43, HCoV-NL63, HCoV-HKU1, and HCoV-229E) collected during the study period in Thailand.

Response:

We have revised the Results section to provide the numbers and proportions of positive samples for all four endemic HCoVs (HCoV-OC43, HCoV-NL63, HCoV-HKU1, and HCoV-229E) for both the overall study period and each individual year. We have also added a new supplementary figure (S2 Fig) illustrating the monthly positivity trends for HCoV-HKU1 and HCoV-229E, which were not previously shown, to complement the existing genotype composition data for HCoV-NL63 and HCoV-OC43 (Fig 1b–1c). All subsequent Supplementary figures have been renumbered accordingly.

The revised Results section now reads:

- Lines 245-249;

"Across the two years combined, 460 HCoV-positive samples were identified from 11,709 ARI hospitalizations screened (3.93%). By species, HCoV-OC43 was the most frequently detected (n = 163, 35.4% of positives), followed by HCoV-NL63 (n = 154, 33.5%), HCoV-HKU1 (n = 77, 16.7%), and HCoV-229E (n = 66, 14.3%)."

- Lines 264-275;

"Monthly genotype composition (Fig 1b–1c) suggested seasonal shifts in dominant lineages. In 2024, HCoV-OC43 accounted for the largest annual share (n = 115, 35.0% of 329 positives that year) and contributed a substantial proportion of detections early in the year, whereas HCoV-NL63 (n = 97, 29.5%) increased and predominated during the middle-to-late months; HCoV-HKU1 (n = 70, 21.3%) and HCoV-229E (n = 47, 14.3%) were detected at lower, fluctuating levels throughout the year (S2 Fig). In 2025, total HCoV detections declined (n = 131); HCoV-NL63 became the most frequently detected species overall (n = 55, 42.0%), increasing in relative contribution during the second half of the year and aligning with the late-season spikes observed in the weekly series, while HCoV-OC43 remained substantial (n = 51, 38.9%) and dominant in the early months, consistent with the early-year wave. HCoV-229E (n = 19, 14.5%) and HCoV-HKU1 (n = 6, 4.6%) contributed smaller proportions in 2025, with monthly trends shown in S2 Fig. "

In addition, please include a more detailed description of the surveillance design, including the sampling strategy, study population, case definitions, and other relevant information.

Response:

We have expanded the Materials and methods section (Lines 117–125) to provide a more detailed description of the surveillance design, including the case definition of ARI, the study population, and the sample collection procedure.

The revised text now reads as follows (Lines 117–125):

"During 2024–2025, we analyzed 11,709 hospitalized acute respiratory infection (ARI) specimens collected at a collaborating tertiary-care hospital in Bangkok, Thailand, as part of an ongoing respiratory virus surveillance program. ARI was defined as a body temperature ≥38°C accompanied by cough, with symptom onset within the preceding 10 days. Patients of all ages presenting with ARI who were hospitalized were eligible for enrollment. Nasopharyngeal swabs were collected in universal transport medium and transported to our center and processed using a routine diagnostic workflow that screens a broad panel of respiratory pathogens by virus-specific real-time RT-PCR assays (e.g., influenza A/B, RSV, SARS-CoV-2, parainfluenza viruses [PIV1–4], adenovirus, and rhinovirus)."

5.3. The manuscript states that a subset of coronavirus-positive specimens was randomly selected for partial spike sequencing (Lines 145–146). However, no further details are provided regarding this “random selection”. The authors should clarify how randomization was implemented, whether sequencing selection was stratified by month, Ct value, hospital, age, or viral species, and whether any unsuccessful sequencing attempts occurred. This information is important, as these factors could introduce bias into genotype composition and seasonal lineage interpretation.

Response:

We appreciate the reviewer's comment for raising this important point. We wis

Attachment Submitted filename: Journal Requirements and reviewer comments.docx

# Peer review: Decision Letter 1

**DOI:** 10.1371/journal.pone.0357483.r003

**Authors:** Haitham Amer

18 Aug 2026

Spatiotemporal dynamics and phylogeography of HCoV-NL63 and HCoV-OC43 in Thailand, 2024–2025

PONE-D-26-07960R1

Dear Dr. Poovorawan,

We’re pleased to inform you that your manuscript has been judged scientifically suitable for publication and will be formally accepted for publication once it meets all outstanding technical requirements.

Within one week, you’ll receive an e-mail detailing the required amendments. When these have been addressed, you’ll receive a formal acceptance letter and your manuscript will be scheduled for publication.

An invoice will be generated when your article is formally accepted. Please note, if your institution has a publishing partnership with PLOS and your article meets the relevant criteria, all or part of your publication costs will be covered. Please make sure your user information is up-to-date by logging into Editorial Manager at Editorial Manager® and clicking the ‘Update My Information' link at the top of the page. For questions related to billing, please contact billing support.

If your institution or institutions have a press office, please notify them about your upcoming paper to help maximize its impact. If they’ll be preparing press materials, please inform our press team as soon as possible -- no later than 48 hours after receiving the formal acceptance. Your manuscript will remain under strict press embargo until 2 pm Eastern Time on the date of publication. For more information, please contact onepress@plos.org.

Kind regards,

Haitham Mohamed Amer, PhD

Academic Editor

PLOS One

Reviewers' comments:

Reviewer's Responses to Questions

**Comments to the Author**

Reviewer #2: All comments have been addressed

Reviewer #3: All comments have been addressed

**********

2. Is the manuscript technically sound, and do the data support the conclusions?

Reviewer #2: Yes

Reviewer #3: Yes

**********

3. Has the statistical analysis been performed appropriately and rigorously?

Reviewer #2: Yes

Reviewer #3: Yes

**********

4. Have the authors made all data underlying the findings in their manuscript fully available?

The PLOS Data policy

Reviewer #2: Yes

Reviewer #3: Yes

**********

5. Is the manuscript presented in an intelligible fashion and written in standard English?

Reviewer #2: Yes

Reviewer #3: Yes

**********

Reviewer #2: The authors have substantially revised the manuscript and have addressed most of the concerns raised in the previous review. The revised manuscript is clearer, particularly regarding the surveillance population, the distribution of the four endemic human coronaviruses, the selection criteria for sequencing, genotype nomenclature, and the limitations associated with the use of partial spike sequences. The correction of the terminology concerning “species” versus “genotype” and the clarification of the HCoV-OC43 genotype nomenclature are also appreciated.

Overall, the study provides useful epidemiological and evolutionary information on HCoV-NL63 and HCoV-OC43 circulation in Thailand during 2024–2025. The integration of routine surveillance, molecular characterization, phylogenetic analysis, and phylogeographic inference represents a strength of the work.

However, some aspects still require careful consideration before the manuscript can be considered fully satisfactory. In particular, the genomic analyses are based exclusively on partial spike sequences, and the sequencing subset was defined using a Ct <30 threshold. Consequently, the genomic dataset may not be fully representative of all HCoV-positive specimens, and this potential selection bias should be more explicitly acknowledged when interpreting genotype distributions, temporal patterns, and phylogeographic relationships. The authors should also avoid interpretations that could imply genome-wide transmission histories or evolutionary patterns.

The phylogeographic results would benefit from additional caution regarding the interpretation of inferred migration routes, particularly given the likely heterogeneity in global sampling intensity. The inferred transitions between Thailand and other geographic regions should be presented as phylogenetic/phylogeographic associations rather than direct evidence of viral movement or epidemiological transmission between countries.

I also recommend that the authors carefully verify the genotype assignments reported in Supplementary Table S7. The correction of the previous nomenclature error is welcome, but assigning corrected genotypes based primarily on matching sample sizes should be independently verified against the phylogenetic trees and the established genotype definitions/reference sequences.

Finally, the manuscript would benefit from a clearer discussion of the limitations associated with the single tertiary-care hospital surveillance setting, the Ct-based sequencing strategy, uneven international sequence availability, and the use of partial spike sequences for recombination and selection analyses. The authors should also ensure that the ethical approval/informed consent or waiver procedures and sequence/data accession information are clearly reported.

Subject to satisfactory clarification of these points and a final careful review of the figures, supplementary tables, genotype annotations, and methodological descriptions, I consider the study potentially suitable for publication. The manuscript addresses an interesting and relevant question and provides valuable data on the post-COVID-19 epidemiology and evolution of endemic human coronaviruses in Thailand.

Reviewer #3: The authors have successfully addressed all substantive concerns raised in the previous review. This manuscript provides valuable baseline genomic and epidemiological data for endemic HCoVs in Southeast Asia during the post-COVID-19 era. The corrected terminology, clarified methodology, and appropriate qualification of partial-sequence limitations significantly strengthen the work.

**********

what does this mean?). If published, this will include your full peer review and any attached files.

If you choose “no”, your identity will remain anonymous but your review may still be made public.

**Do you want your identity to be public for this peer review?**  For information about this choice, including consent withdrawal, please see our Privacy Policy

Reviewer #2: No

Reviewer #3: **Yes:**  Mohamed A. Farrag

**********

# Editor report: Acceptance letter

**DOI:** 10.1371/journal.pone.0357483.r004

**Authors:** Haitham Amer

PONE-D-26-07960R1

PLOS One

Dear Dr. Poovorawan,

I'm pleased to inform you that your manuscript has been deemed suitable for publication in PLOS One. Congratulations! Your manuscript is now being handed over to our production team.

At this stage, our production department will prepare your paper for publication. This includes ensuring the following:

* All references, tables, and figures are properly cited

* All relevant supporting information is included in the manuscript submission,

* There are no issues that prevent the paper from being properly typeset

You will receive further instructions from the production team, including instructions on how to review your proof when it is ready. Please keep in mind that we are working through a large volume of accepted articles, so please give us a few days to review your paper and let you know the next and final steps.

Lastly, if your institution or institutions have a press office, please let them know about your upcoming paper now to help maximize its impact. If they'll be preparing press materials, please inform our press team within the next 48 hours. Your manuscript will remain under strict press embargo until 2 pm Eastern Time on the date of publication. For more information, please contact onepress@plos.org.

You will receive an invoice from PLOS for your publication fee after your manuscript has reached the completed accept phase. If you receive an email requesting payment before acceptance or for any other service, this may be a phishing scheme. Learn how to identify phishing emails and protect your accounts at https://explore.plos.org/phishing.

If we can help with anything else, please email us at customercare@plos.org.

Thank you for submitting your work to PLOS One and supporting open access.

Kind regards,

PLOS One Editorial Office Staff

on behalf of

Dr. Haitham Mohamed Amer

Academic Editor

PLOS One
